# Comprehensive analysis of genetic and evolutionary features of the hepatitis E virus

**DOI:** 10.1186/s12864-019-6100-8

**Published:** 2019-10-29

**Authors:** Sarra Baha, Nouredine Behloul, Zhenzhen Liu, Wenjuan Wei, Ruihua Shi, Jihong Meng

**Affiliations:** 1 Department of Gastroenterology, Zhongda Hospital, Southeast University, Jiangsu Province, China; 2 College of Basic Medicine, Shanghai University of Medicine & Health Sciences, Shanghai, China

**Keywords:** Hepatitis E virus, Codon usage, Natural selection, Bayesian phylogenetics, Evolution

## Abstract

**Background:**

The hepatitis E virus (HEV) is the causative pathogen of hepatitis E, a global public health concern. HEV comprises 8 genotypes with a wide host range and geographic distribution. This study aims to determine the genetic factors influencing the molecular adaptive changes of HEV open reading frames (ORFs) and estimate the HEV origin and evolutionary history.

**Results:**

Sequences of HEV strains isolated between 1982 and 2017 were retrieved and multiple analyses were performed to determine overall codon usage patterns, effects of natural selection and/or mutation pressure and host influence on the evolution of HEV ORFs. Besides, Bayesian Coalescent Markov Chain Monte Carlo (MCMC) Analysis was performed to estimate the spatial-temporal evolution of HEV. The results indicated an A/C nucleotide bias and ORF-dependent codon usage bias affected mainly by natural selection. The adaptation of HEV ORFs to their hosts was also ORF-dependent, with ORF1 and ORF2 sharing an almost similar adaptation profile to the different hosts. The discriminant analysis based on the adaptation index suggested that ORF1 and ORF3 could play a pivotal role in viral host tropism.

**Conclusion:**

In this study, we estimate that the common ancestor of the modern HEV strains emerged ~ 6000 years ago, in the period following the domestication of pigs. Then, natural selection played the major role in the evolution of the codon usage of HEV ORFs. The significant adaptation of ORF1 of genotype 1 to humans, makes ORF1 an evolutionary indicator of HEV host speciation, and could explain the epidemic character of genotype 1 strains in humans.

## Background

Hepatitis E virus (HEV), a member of the genus Orthohepevirus in the family Hepeviridae, is a non-enveloped positive-sense RNA virus, with a full-length genome of 7.2 kb [1]. The HEV genome is composed of 3 open reading frames (ORF) [2]. The ORF1 encodes for a non-structural polyprotein of 1693 amino acids (aa) [3]; the ORF2 encodes the viral structural capsid protein of 660aa which is responsible for virion assembly [4], and the ORF3 that overlaps ORF2 and encodes a small phosphoprotein of 114aa associated with virion morphogenesis and release as well as other interactions with host cell components [5]. Since its discovery as the causative agent of an epidemic non-A, non-B hepatitis in Kashmir, India in 1978 [6], the list of HEV isolates keeps growing along with the list of its hosts. HEV is a global public health threat causing both epidemics and sporadic cases of acute hepatitis [7, 8].

The recent classification proposed by Smith et al. [9] groups the HEV isolates into eight genotypes: genotypes 1 and 2 are transmitted fecal-orally between humans; genotypes 3 and 4 circulate in animal populations and can be transmitted to humans zoonotically from infected pigs, deer, and wild boar; genotypes 5 and 6 were identified in Japanese wild boars; finally, genotypes 7 and 8 are novel genotypes identified in camels [10]. Further, Smith et al. expanded the initial work of Lu et al. [11] and divided the HEV genotypes into subtypes by the analysis of nucleotide *p*-distances of all available complete HEV genome sequences and assigned reference sequences for each subtype [9].

All amino acids, except methionine (Met) and tryptophan (Trp), are coded by more than one synonymous codon. However, synonymous codons are not randomly selected within and between genomes. Such preference of one synonymous codon over others is commonly known as codon usage bias [12]. This phenomenon has been observed in a wide range of organisms, from prokaryotes to eukaryotes and viruses. There are two main forces that affect usage of synonymous codons: the mutational bias which refers to the asymmetric occurrence of mutations, and natural selection for favored specific synonymous codon usage patterns associated with specific gene functions. These two types of mechanisms are not mutually exclusive, and both are useful for understanding the evolutionary phenomena occurring within and between species (in our case within and between HEV genotypes).

The study of codon usage patterns can provide useful insights into the molecular evolution, extend our understanding of the regulation of viral gene expression, and improve vaccine design, for which the efficient expression of viral proteins may be required to generate efficient immune responses. Besides, A Bayesian statistical inference approach have been recently developed and used for the estimation of viruses’ origins and the reconstruction of their temporal and spatial dispersion [13]. Therefore, given the continuously growing number of the reported HEV genome sequences, in this study, we performed an up to date comprehensive analysis of the composition and codon usage features of HEV full-genomes reported between 1982 and 2017, followed by Bayesian phylogenetics analysis to retrace the evolutionary history of HEV.

## Results

### Nucleotide composition of HEV ORFs

To determine the potential impact of nucleotide constraints on codon usage, the values of nucleotide contents in all individual HEV coding sequences (ORF1, 2, and 3) were determined (Table 1 and Additional file 2: Table S2). The results revealed that nucleotide A was under-represented with an average of 18.36 ± 0.6%, 17.99 ± 0.5%, 11.19 ± 0.74% in ORF1, ORF2 and ORF3 respectively; whereas C was over-represented with an average of 28.88 ± 1.14%, 30.93 ± 1.2%, 38.8 ± 0.93% in ORF1, ORF2 and ORF3, respectively. However, nucleotides G and T (U) were distributed at random. All HEV coding sequences showed an overall GC content value exceeding 50%, with the highest content observed in ORF3 (67%), showing thus, a weak compositional bias in favor of G + C. In addition, the GC content at the different codon position was not uniformly distributed between the ORFs: in ORF1 and ORF2, the GC content was higher at the first codon position (62.21% ± 0.55, 60.6% ± 0.93 respectively), whereas in ORF3 the GC content was higher at the third codon position (72.69% ± 2.1). To further analyze the potential role of nucleotide content in shaping the codon usage patterns in the HEV genes, the codon composition at the third position (A3, U3, G3, and C3) were calculated. The results indicated that in ORF1 and ORF2, U and C ending codons were preferred over A and G ending ones; while in ORF3, C and G ending codons were more represented than A and U ending ones.

**Table 1:** Nucleotide composition of the HEV ORFs

	ORF1	ORF2	ORF3
	Average (Std. D)	Average (Std. D)	Average (Std. D)
A	18.36 (0.63)	18.00 (0.47)	11.20 (0.75
C	28.88 (1.14)	30.93 (1.21)	38.83 (0.94
T	25.86 (0.69)	26.90 (1.17)	21.78 (0.73
G	26.90 (0.35)	24.17 (0.45)	28.20 (0.58
A3	11.72 (1.63)	10.19 (1.05)	10.88 (1.51
C3	30.45 (2.99)	29.88 (2.93)	39.89 (1.60
T3	32.27 (1.80)	38.37 (3.22)	16.42 (1.68
G3	25.56 (0.91)	21.57 (1.17)	32.81 (1.31
GC	55.78 (1.13)	55.10 (1.44)	67.02 (1.12
GC1	62.21 (0.56)	60.61 (0.93)	66.58 (2.37
GC2	49.12 (0.41)	53.24 (0.35)	61.79 (1.85
GC3	56.01 (2.98)	51.45 (3.54)	72.70 (2.10)

*Std. D* standard deviation. The values are represented as percentage

### RSCU patterns of the HEV coding sequences

To determine the codon usage patterns and preferences for synonymous codons in the HEV coding sequences, the RSCU values were computed for every codon in each ORF sequence. Codons with an RSCU value of > 1.6 were considered over-represented, whereas codon with an RSCU value of < 0.6 was considered under-represented. The results are shown in Table 2, Additional file 3: Table S3 and Table S4. Among the 18 most abundantly used codons, the U/C ended codons were preferred in ORF1s and ORF2s while the C/G ended ones were preferred in the ORF3s when the HEV coding sequences were not differentiated according to their genotypic group.

**Table 2:** RSCU patterns of the HEV ORFs

Amino acid	Codon	ORF1		ORF2		ORF3	
		Mean	SD	Mean	SD	Mean	SD
Phe	UUU	**1.16**	0.12	**1.01**	0.19	0.62	0.40
	UUC	0.84	0.12	0.99	0.19	**1.38**	0.40
Leu	UUA	0.45	0.15	0.39	0.17	0.01	0.05
	UUG	0.90	0.18	0.99	0.30	0.76	0.34
	CUU	**1.63**	0.23	**1.95**	0.29	0.61	0.31
	CUC	1.32	0.27	1.19	0.28	1.47	0.54
	CUA	0.51	0.11	0.34	0.15	0.85	0.37
	CUG	1.19	0.15	1.14	0.28	**2.29**	0.43
Ile	AUU	**1.37**	0.14	**1.53**	0.30	1.02	0.30
	AUC	0.95	0.17	0.95	0.25	**1.03**	0.20
	AUA	0.68	0.14	0.52	0.20	0.95	0.35
Val	GUU	**1.44**	0.15	**1.77**	0.24	0.59	0.20
	GUC	1.14	0.16	1.18	0.23	1.49	0.35
	GUA	0.32	0.11	0.26	0.14	0.20	0.23
	GUG	1.10	0.15	0.79	0.19	**1.71**	0.38
Ser	UCU	**1.67**	0.23	**2.39**	0.39	0.83	0.29
	UCC	1.32	0.25	1.57	0.27	0.86	0.35
	UCA	0.85	0.20	0.71	0.23	0.34	0.40
	UCG	0.80	0.18	0.70	0.16	**1.98**	0.35
	AGU	0.67	0.15	0.32	0.11	0.31	0.28
	AGC	0.69	0.18	0.31	0.13	1.68	0.31
Pro	CCU	**1.39**	0.14	**1.25**	0.21	0.75	0.15
	CCC	1.12	0.15	1.19	0.19	1.16	0.33
	CCA	0.69	0.15	0.63	0.13	0.52	0.16
	CCG	0.80	0.13	0.92	0.15	**1.57**	0.33
Thr	ACU	1.23	0.20	**1.59**	0.28	0.05	0.21
	ACC	**1.40**	0.27	1.31	0.31	**2.68**	0.71
	ACA	0.82	0.14	0.71	0.17	0.95	0.51
	ACG	0.55	0.13	0.39	0.12	0.32	0.48
Ala	GCU	1.21	0.10	**1.69**	0.25	0.43	0.24
	GCC	**1.60**	0.21	1.55	0.23	**1.87**	0.34
	GCA	0.59	0.14	0.35	0.12	0.55	0.25
	GCG	0.60	0.12	0.42	0.12	1.15	0.34
Tyr	UAU	**1.07**	0.16	**1.31**	0.17	**0.40**	0.80
	UAC	0.93	0.16	0.69	0.17	0.15	0.53
His	CAU	**1.10**	0.13	**1.28**	0.27	0.25	0.35
	CAC	0.90	0.13	0.72	0.27	**1.75**	0.35
Gln	CAA	0.37	0.12	0.43	0.13	**1.12**	0.44
	CAG	**1.63**	0.12	**1.57**	0.13	0.88	0.44
Arn	AAU	**1.10**	0.15	**1.31**	0.19	0.88	0.70
	AAC	0.90	0.15	0.69	0.19	**0.89**	0.70
Lys	AAA	0.60	0.16	0.65	0.31	0.00	0.00
	AAG	**1.40**	0.16	**1.35**	0.31	**0.01**	0.16
Asp	GAU	**1.13**	0.12	**1.14**	0.18	0.82	0.70
	GAC	0.87	0.12	0.86	0.18	**1.18**	0.70
Glu	GAA	0.41	0.09	0.38	0.14	0.17	0.55
	GAG	**1.59**	0.09	**1.62**	0.14	**1.48**	0.88
Cys	UGU	0.95	0.17	0.70	0.41	0.59	0.22
	UGC	**1.05**	0.17	**1.30**	0.41	**1.41**	0.22
Arg	CGU	1.61	0.28	2.03	0.37	1.44	0.66
	CGC	**1.81**	0.36	**2.37**	0.33	**3.01**	0.63
	CGA	0.42	0.16	0.44	0.15	0.19	0.36
	CGG	1.28	0.34	0.88	0.20	1.22	0.45
	AGA	0.23	0.13	0.05	0.07	0.01	0.08
	AGG	0.65	0.10	0.23	0.22	0.13	0.29
Gly	GGU	1.15	0.20	**1.62**	0.23	0.15	0.25
	GGC	**1.70**	0.23	1.42	0.20	1.54	0.22
	GGA	0.21	0.08	0.21	0.11	0.22	0.25
	GGG	0.94	0.11	0.75	0.15	**2.08**	0.27

The over-representedcodons are indicated in bold

Further, the RSCU genotype-specific patterns have been analyzed and the results showed that the preferred codons varied among the different genotypes. The common and uncommon preferred codons in the three ORFs among the eight HEV genotypes are shown in Tables S3 and S4. More codon over-representation was observed in the ORF3s, followed by ORF2s and finally ORF1s with the lowest number of over-represented codons, and this pattern was common for the eight genotypes. Interestingly, the genotype 1 isolates showed the highest number of over-represented preferred codons in the different ORFs: 9, 10 and 11 in ORF1, ORF2 and ORF3, respectively.

The genotype-specific RSCU patterns highlight the independent evolutionary dynamics of the HEV isolates. In line with compositional analysis, the RSCU analysis confirmed the comparatively higher codon usage bias towards U/C ended codons in ORF1 and ORF2; and towards C/G ended codons in ORF3.

### Correspondence analysis of the RSCU variations in the HEV ORFs

**Fig. 1:**
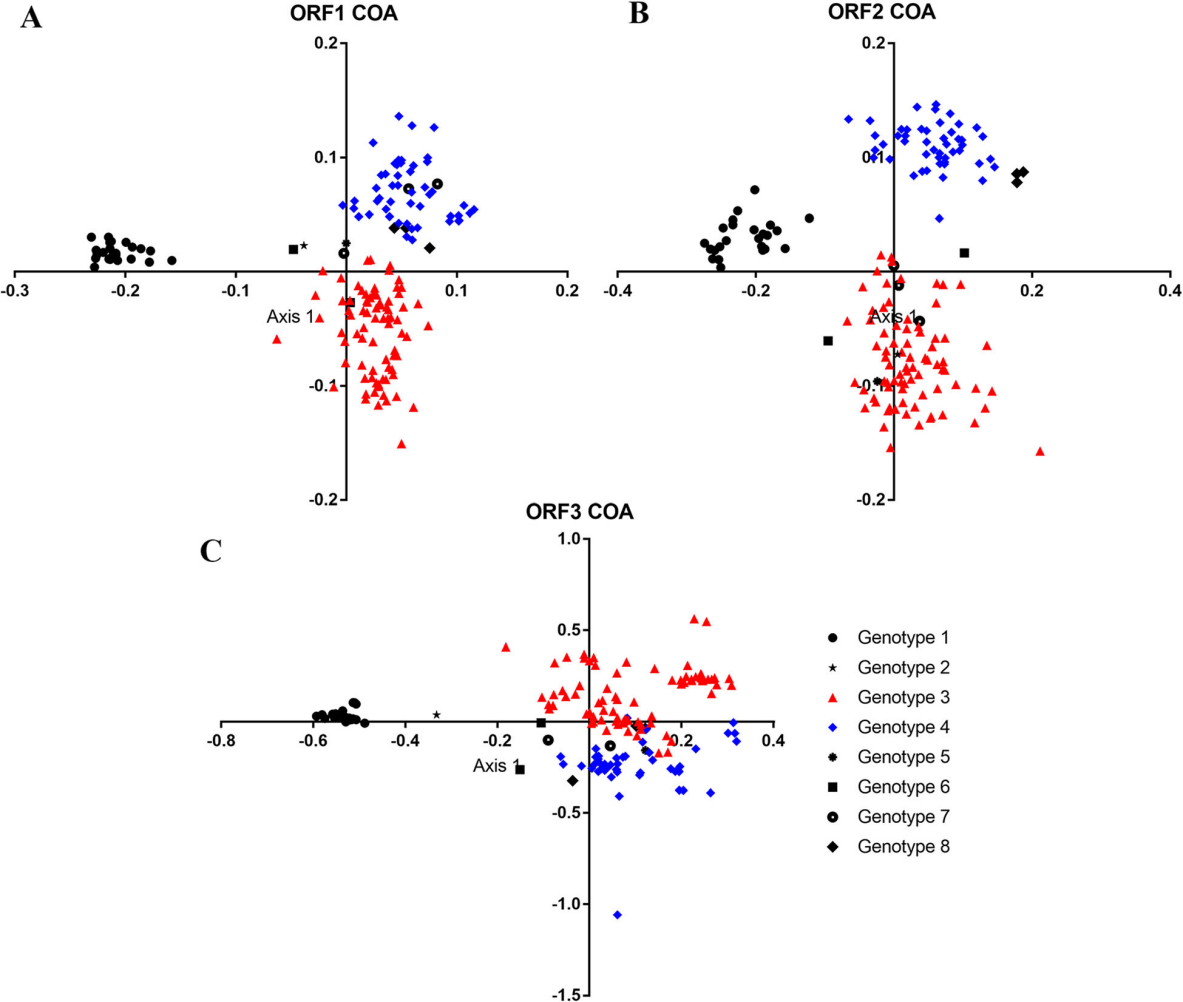
Correspondence analysis (CA) based on the relative synonymous codon usage (RSCU). Genotype-specific CA plots were constructed for HEV ORF1, 2 and 3 (**a**, **b** and **c**, respectively)

To investigate synonymous codon usage variation, correspondence analysis (COA), a multivariate statistical method, was executed on the RSCU values of HEV coding sequences. The results revealed that the first and second principal axes accounted for the majority of the data inertia (ORF1: ƒ´1 = 27.5%, ƒ´2 = 12.65%, ORF2: ƒ´1 = 19.63%, ƒ´2 = 13.96%, ORF3: ƒ´1 = 15.93%, ƒ´2 = 12.5%), indicating that ƒ´1 and ƒ´2 axes explains the major proportion of codon usage variations. The COA analysis built on RSCU of codons also revealed that the codon usage patterns of HEV genotypes were different and ORF-dependent. The HEV genotypes had different codon usage biases. For ORF1 and ORF2, HEV strains of genotype 1, 3 and 4 were grouped into three well-defined clusters on the axes plots, whereas the HEV strains for other genotypes were distributed within or between genotype 3 and 4 clusters (Fig. 1a and B). However, the distribution of these other genotypes (2, 5, 6, 7 and 8) should be interpreted carefully given the very low number of sequences available (1, 1, 2, 3 and 3 sequences, respectively). Furthermore, the clustering of genotype 1, 3 and 4 strains was very consistent with the phylogenetic classification of the HEV complete genome reported by Smith et al. [1]. On the other hand, the analysis of ORF3s showed that the HEV strains were grouped into only two clusters: a cluster composed of HEV genotype 1 and 2 strains, and a cluster of the remaining strains, indicating that the RCSU values of ORF3s allow the distinction between human HEV genotypes and zoonotic genotypes (H and Z genotypes) (Fig. 1c).

### The variation of the effective number of codons among the HEV ORFs

**Fig. 2:**
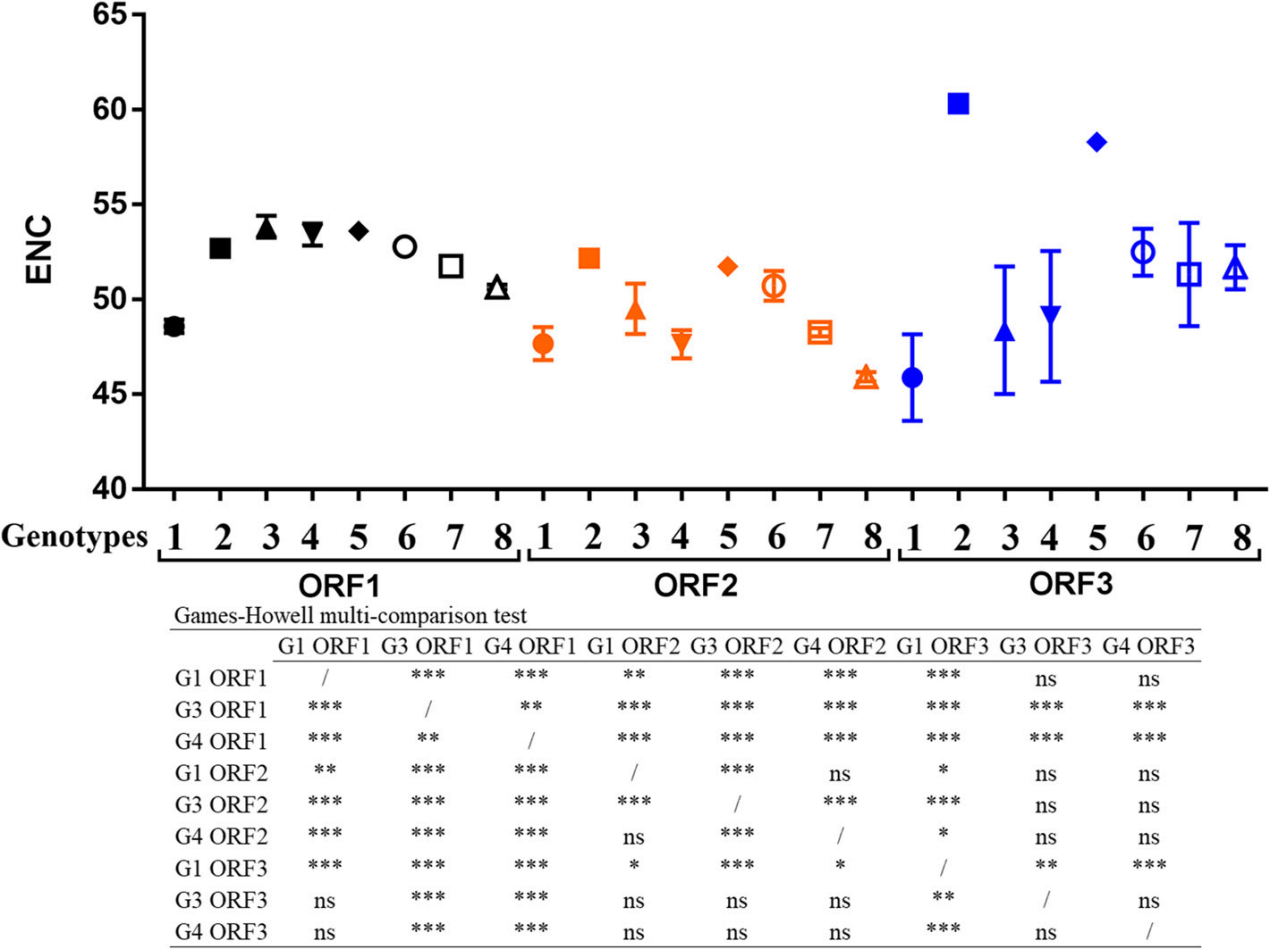
Genotype-specific comparative analysis of ENC values of three HEV ORFs coding sequences. The data are presented as mean ± standard error; *p < 0.05, **p < 0.01, ***p < 0.001; ns: non-significant *p* > 0.05

To estimate the degree of the codon usage bias within the three HEV ORFs, the ENC values were computed. Regardless of the genotype, an overall mean value of 52.8 ± 1.91, 48.62 ± 1.5, and 48.5 ± 3.6 were obtained for ORF1, ORF2, and ORF3 respectively. No significant difference was observed between the ORF2s and ORF3s. However, the ORF1s displayed significantly higher ENC values. Further, the analysis of the ENC between the different genotypes revealed, as shown in Fig. 2**,** a significant difference in the overall ENC distribution between the three ORFs according to the genotype, as determined by one-way ANOVA (*p* < 0.001), the Welsh test (*p* < 0.001) and Brown-Forsythe test (*p* < 0.001).

Concerning ORF1, genotype 1 has the lowest ENC values, whereas genotype 3 has the highest values. Concerning ORF2, Genotype 8 displayed the lowest ENC, whereas genotype 2 displayed the highest one. In comparison to ORF1, an overall decrease in ENC value was observed for all genotypes especially for genotypes 3 and 4. Finally, for the ORF3s, the lowest ENC was found in genotype 1 sequences, whereas the highest one was observed in genotype 2. Interestingly, the genotype 2 ORFs displayed higher ENC than the other genotypes, but these results should be taken carefully since only one genotype 2 strain was available for the study.

The multi-comparison of the ENC values between the ORFs of genotypes 1, 3 and 4 revealed that all the differences were statistically significant except between the ORF2 of genotype 1 and the ORF2 of genotype 4; and when the ORF3s of genotypes 3 and 4 were compared together or when compared to the ORF1 of genotype 1 or the ORF2 of genotype 3 (Fig. 2).

Overall, the mean ENC values suggested a relatively significant difference and genotype-specific evolution of codon usage bias within individual HEV coding sequences.

### Correlation analysis

The correlation of different nucleotides content with the two principal axes of COA was performed:

1. For ORF1, the first axis had a significant positive correlation with A3 (*r* = 0.664, *p* < 0.01), U3 (*r* = 0.808, *p* < 0.01) and a significant negative correlation with C3(r = − 0.794, *p* < 0.01), GC3 (*r* = − 0.876, *p* < 0.01); the second axis had a positive correlation with U3 (r = − 0.418, *p* < 0.01), G3 (*r* = − 0.204, *p* < 0.01) and negative correlation with C3 (*r* = − 0.449, *p* < 0.01), GC3(*r* = − 0.305, *p* < 0.01); there was also a significant negative correlation between the ENC and GC3s (*r* = − 0.261, *p* < 0.0001), and the ENC value had a positive (*r* = 0.401, *p* < 0.01) and negative (*r* = − 0.375, *p* < 0.01) correlations with the first and second axes, respectively.
2. For ORF2, the fist axis had a positive correlation with A3 (*r* = 0.333, *p* < 0.01), U3 (*r* = 0.651, *p* < 0.01) and significant negative correlation with C3(*r* = − 0.715, *p* < 0.01), G3(*r* = − 0.341, *p* < 0.01), GC3 (*r* = − 0.671, *p* < 0.01), while the second axis had a significant negative correlation with A3 (*r* = − 0.208, *p* < 0.01), C3(*r* = − 0.311, *p* < 0.01), G3(*r* = − 0.553, *p* < 0.01), GC3(*r* = − 0.450, *p* < 0.01), and ENC (*r* = − 0.567, *p* < 0.01); and a positive correlation with U3 (*r* = − 0.462, *p* < 0.01).
3. However, in the case of ORF3 was slightly different, the first axis had only a significant positive and negative correlation with U3 (*r* = 0.273, *p* < 0.01) and A3 (*r* = − 0.372, *p* < 0.01), respectively; whereas the second axis had a significant negative correlation with C3 (*r* = − 0.349, *p* < 0.01), G3 (*r* = − 0.292, *p* < 0.01), GC3 (*r* = − 0.449, *p* < 0.01) and ENc (*r* = − 0.173, *p* < 0.05).

Overall, these results demonstrated that the compositional constraints indeed affect the codon usage bias in all HEV coding sequences, with a different magnitude and in an ORF-dependent manner.

### Codon usage adaptation of the HEV ORFs to different hosts

The CAI values range from 0 to 1, being 1 if the frequency of codon usage by the virus equals the frequency of codon usage of the reference set. In HEV ORF1s, ORF2s and ORF3s, the highest CAI was noted in relation to *Macaca fascicularis* (0.79 ± 0.01, 0.78 ± 0.01, 0.071 ± 0.02), followed by *Homo sapiens* (0.73 ± 0.01, 0.72 ± 0.01, 0.69 ± 0.02), *Camelus bactrianus* (0.7 ± 0.01, 0.67 ± 0.01, 0.67 ± 0.01), *Macaca muluta* (0.67 ± 0.01, 0.66 ± 0.01, 0.67 ± 0.01), *Sus scrofa* (0.65 ± 0.02, 0.63 ± 0.01, 0.65 ± 0.02), *Camelus dromedaries* (0.63 ± 0.02, 0.61 ± 0.01, 0.63 ± 0.02), *Oryctolagus cuniculus* (0.61 ± 0.02, 0.59 ± 0.01, 0.63 ± 0.02) and finally *Sus scrofa domestica* (0.55 ± 0.01, 0.53 ± 0.01, 0.57 ± 0.03).

Furthermore, to validate the observed difference in the adaptation index and to provide statistical support to CAI analysis, the expected CAI (E-CAI) and normalized CAI (N-CAI) were calculated for the three HEV ORFs in relation to the eight hosts included in this study. The E-CAI server calculates the expected value of the CAI by generating 500 sequences that have similar nucleotide content and amino acid composition as the sequence of interest (in this case a given HEV ORF sequence), and then, a Kolmogorov–Smirnov test was applied to confirm that the generated random sequences show a normal distribution. The E-CAI values were used to discern whether the differences in CAI are statistically significant and arise from the codon preferences or whether they are just artifacts related to the internal biases in the G + C composition and/or amino acid composition of the query sequences. The normalized CAI, which is defined as the quotient between the CAI of a gene and its E-CAI is an effective way to compare the adaptation of codon usage of a gene to a given host. An N-CAI value greater than 1 indicates that the adaptation process in the codon usage is statistically significant and independent of the nucleotide and amino acid composition [14].

Interestingly, the results showed that the adaptation index was ORF-dependent (Additional file 4: Table S5). Regardless of the genotype, the ORF1 was significantly well adapted to *Macaca fascicularis* codon usage (N-CAI = 1.006 ± 0.01), whereas ORF2 was significantly adapted to *Homo sapiens* (N-CAI = 1.0048 ± 0.01) and *Macaca fascicularis* (N-CAI = 1.003 ± 0.01). No significant adaption was noted for ORF3 in relation to all hosts.

**Fig. 3:**
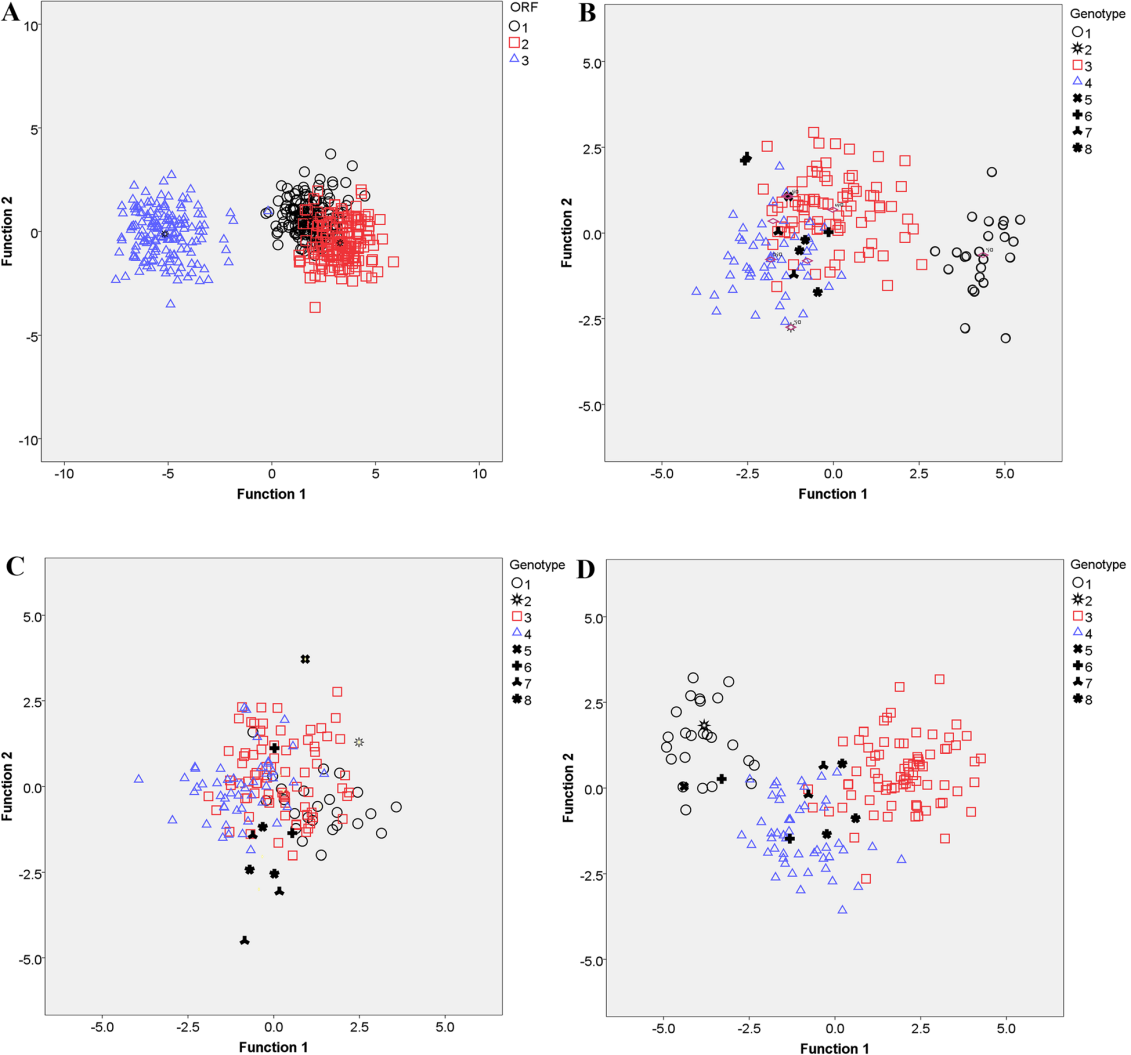
Discriminant analysis based on the normalized codon adaptation index (N-CAI) of the HEV ORFs in relation to all the hosts. All three HEV ORFs were analyzed together regardless of the genotype and the data were colored according to the ORF (**a**). Then, the ORF1s, 2 s and 3 s were analyzed separately and the data were colored according to the different genotypes (**b**, **c** and **d**, respectively)

Furthermore, a discriminant analysis was performed to highlight the difference in N-CAI between the three HEV ORFs in relation to all the hosts. As shown in Fig. 3, ORF1 and ORF2 sequences are clustered together and form a single group, well separated from the ORF3 sequences, indicating that ORF1 and ORF2 genes have an almost similar adaptation profile to the different hosts (Fig. 3a and Additional file 5: Table S6). Concerning the genotype-specific pattern of the N-CAI (Fig. 3b, c, d, and Additional file 5: Table S6), the results showed that for ORF2 sequences, no discriminant separation of the HEV strains was observed. On the other hand, however, a clear separation into two clusters were observed for ORF1 and ORF3 sequences: for ORF1s, the first cluster contained HEV strains belonging to genotype 1 and the second cluster contained all the other remaining HEV strains; whereas for ORF3s, genotype 1, 2 strains along with single genotype 5 and 6 strains were grouped together, and the remaining strains formed the second cluster. It is worth noting that the clustering shown in Fig. 3b and d is in accordance with the classification of HEV strains into human genotypes and zoonotic genotypes, which suggests that codon adaptation could play a pivotal role in viral host tropism as well as the severity of the infection (the epidemic character of the HEV genotype 1 infections).

### Similarity analysis between the codon usage bias of the HEV ORFs and the HEV hosts

To determine the potential influence of the codon usage patterns of the main hosts on the evolution of the codon usage patterns of HEV coding sequences, a similarity analysis was conducted. In this method, each one of the 59 synonymous codons is taken into account and analyzed all together to estimate the similarity of the overall codon usage patterns between HEV and its host, rather than one to one codon comparison. The results showed that in comparison to all hosts, the ORF3 had the highest degree of similarity followed by ORF2 and ORF1, with the strongest similarities of the three ORFs registered with *Sus scrofa domestica.* When analyzed by genotype, *Sus scrofa domestica* was also found to have the highest similarity degree with the different ORFs in all HEV genotypes, implying that the codon usage patterns of all HEV genotypes have been strongly influenced by *Sus scrofa domestica* (Additional file 6: Figure S1).

### Effects of natural selection versus mutation pressure in shaping the codon usage patterns of HEV ORFs

To determine whether the codon usage patterns of the HEV ORFs sequences have been shaped solely by mutation pressure, natural selection or both, ENC–GC3 plots, neutrality plot and parity rule plot were constructed.

#### ENC-GC3 plot

**Fig. 4:**
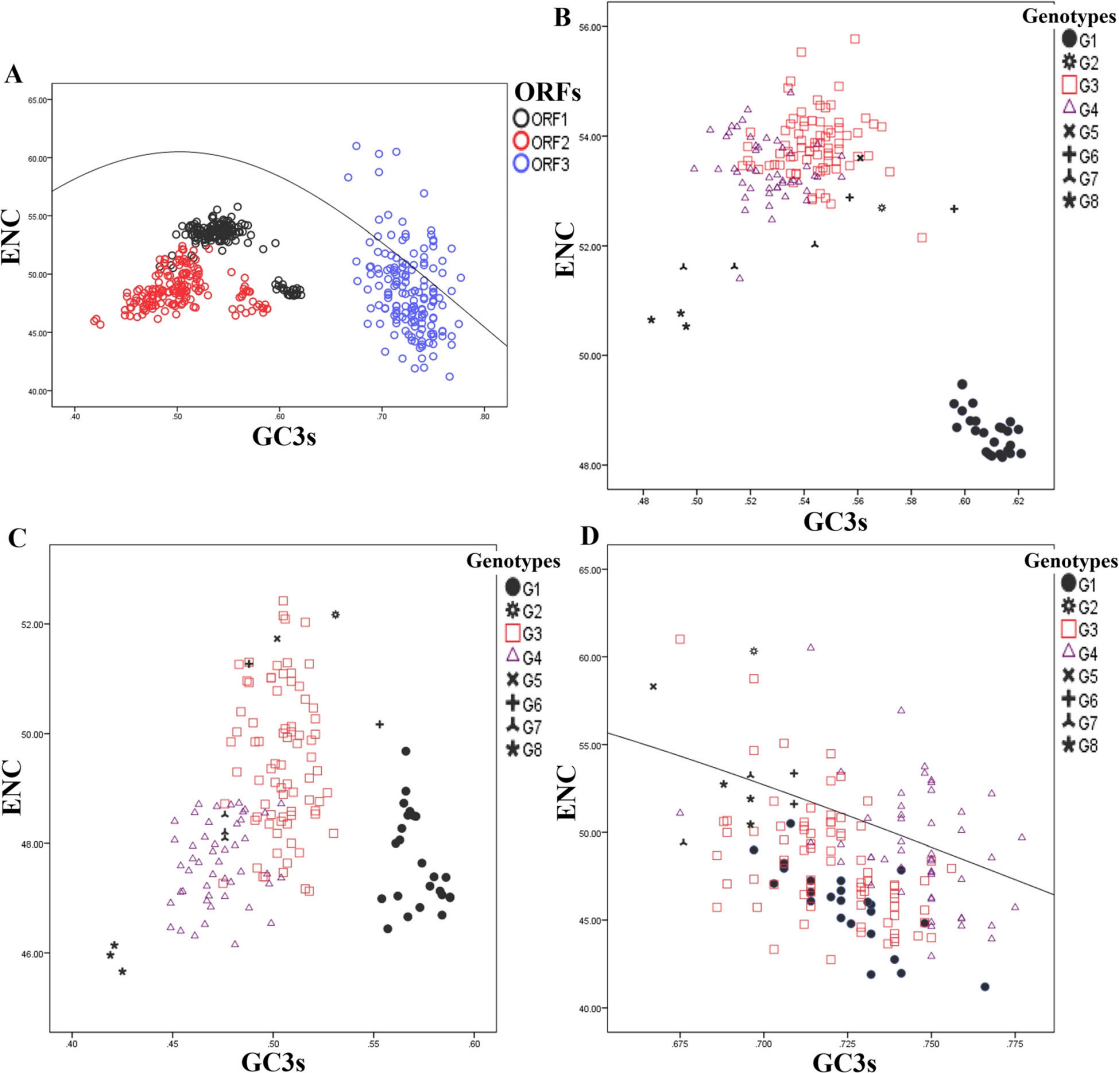
ENC–GC3 plots displaying the relationship between GC3s and effective number of codon (ENC) for three HEV ORFs. All three HEV ORFs were analyzed together regardless of the genotype and the data were colored according to the ORF (**a**). Then, the ORF1s, 2 s and 3 s were analyzed separately and the data were colored according the different genotypes (**b**, **c** and **d**, respectively). The solid curve indicates the expected codon usage if GC compositional constraints alone justify the codon usage bias

The effective number of codons ENC was plotted against the percentage of GC at the third codon position GC3s for each of the three HEV ORFs separately (Fig. 4). In the plot of all HEV ORF1 and ORF2 sequences, HEV strains from all genotypes lay below the null curve considerably. This below-curve position indicates the influence of natural selection in the codon usage pattern of HEV ORF1 and ORF2. However, the effects of mutation pressure and natural selection on individual coding sequences varied in a genotype-specific manner and even within a single strain (Fig. 4b and c). On the other hand, the influence of mutation pressure was not completely absent in HEV ORF3, some coding sequences of genotypes 3, 4 and 7 fell on the expected curve, and other sequences were fallen closely below the curve, showing the dominant influence of mutation pressure rather than natural selection (Fig. 4d).

#### Parity rule plot

To further determine whether the biased codon choices were limited to highly biased protein-coding genes, the relationship between purines (A and G) and pyrimidines (C and T) contents in the four-fold degenerate codon families (Ala, Arg, Gly, Leu, Pro, Ser, Thr and Val) were analyzed with a PR2 plot. The results show that U and C were used more frequently than G and A in the fourfold degenerate codon families in all HEV ORFs, regardless of the genotype (Additional file 7: Figure S2). This unequal use of nucleotides suggests the overlapping influences of natural selection and mutation pressure on the codon preferences in individual HEV coding sequences.

#### Neutrality plots

Next, the neutrality plots P12 (GC1, 2S) versus P3 (GC3s) were constructed to determine the role of mutation-selection equilibrium in shaping the codon usage bias. The results showed that there is a significant positive correlation between the GC12 and GC3 values in ORF1 (*r* = 0.46, *p* < 0.0001), ORF2s (*r* = 0.61, *p* < 0.0001) and ORF3 (*r* = 0.20, *p* < 0.05). However, the slopes of the regression line in ORF1, ORF2, and ORF3 were calculated to be 0.047, 0.093, and 0.082, respectively (Additional file 8: Figure S3), indicating that the influence of direct mutation pressure on the codon usage bias in ORF1, ORF2, and ORF3 was only 4.7, 9.3, and 8.2%, respectively. In contrast, the influence of natural selection in codon usage bias was very high, 95.3, 90.7 and 91.8% in ORF1, ORF2, and ORF3 sequences. Despite the observed correlation in the HEV ORFs, natural selection emerged as the dominant factor influencing the codon usage bias. In HEV ORF1 and ORF2, the correlation between P12 and P3 was not significant (*P* > 0.05) in all genotypes. A significant correlation was observed for ORF3 sequences in genotypes 1 and 3, with a slope of the regression line of 0.29 and 0.122, giving a mutation pressure rate of 2.9 and 1.2%, and a natural selection rate of 97.1 and 98.8%, respectively. Overall, these results indicated the dominant influence of natural selection on the codon usage patterns of three HEV coding sequences in all genotypes.

### Bayesian phylogeny

Using 183 HEV sequences (Additional file 1: Table S1), the tMRCAs for all HEV genotypes were calculated, including the tMRCA for genotypes 1, 3 and 4 individually, as well as for the genotypes 1 and 2, the genotypes 3 and 4 and the four-genotypes together. The mean tMRCAs and the 95% highest posterior probability density as calculated with a coalescent constant size tree prior and a strict clock or relaxed uncorrelated clock models are shown in Table 3.

**Table 3:** Calculated tMRCAs values for HEV partial ORF1 (852 nt at the 3′ end)

tMRCA	Strict clock		Relaxed uncorrelated clock			
			Lognormal		Exponential	
	Mean	95% HPD	Mean	95% HPD	Mean	95% HPD
Genotype clades						
1, 2, 3 and 4	782.26	617.34–952.01	752.37	539.76–995.17	643.212	322.24–1044.84
1 and 2	528.66	397.69–675.96	504.74	311.71–713.90	341.627	133.62–683.44
3 and 4	718.1	569.82–863.28	662.37	461.54–871.85	549.387	273.75–905.95
1	97.96	82.12–114.74	106.80	79.28–137.49	121.726	66.23–197.544
3	247.12	196.69–299.17	264.73	181.02364.46	259.124	117.84–450.49
4	156.88	130.49–182.22	160.96	120.04–204.18	144.828	77.24–226.75
Evolutionary rate (×10 ^−3^ sub/site/year)	1.14	1–1.3	1.28	0.89–1.66	1.76	1.14–2.39

**Fig. 5:**
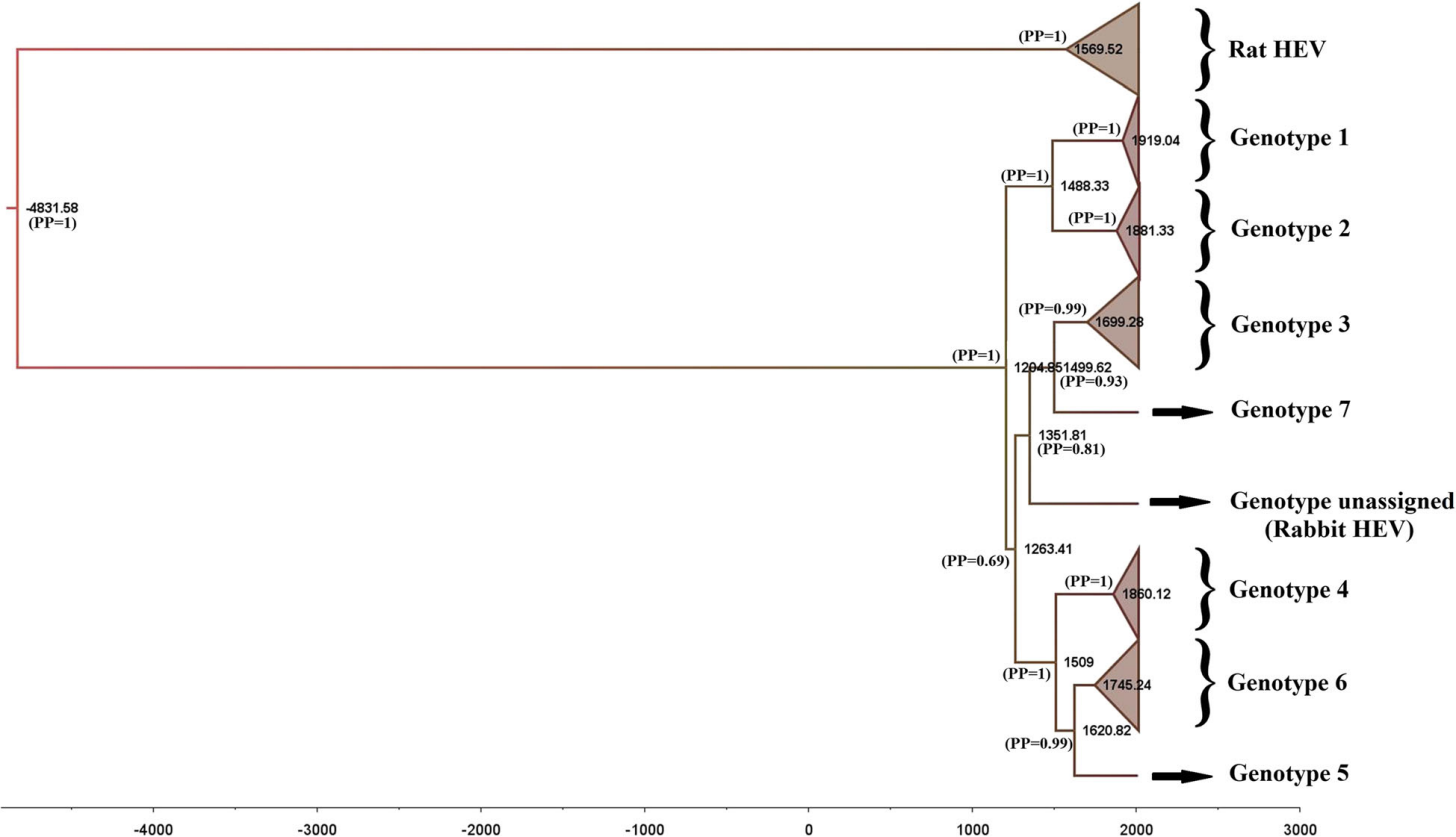
Bayesian phylogenetic maximum clade credibility (MCC) tree for 183 sequences of HEV ORF1 (852 nt of the 3′ end). This tree was constructed using a strict clock model with a constant growth prior. The numbers at each tree represent the mean values for age of the most recent common ancestor (MRCA) at that node (PP = posterior probability). Each genotype clade was collapsed and labeled (refer to Additional file 9: Figure S4 for the detailed tree with each sequence labeled with its year of collection followed by GenBank accession number, genotype, region of isolation and the host)

The data from the analysis of the 3′ end of HEV ORF1 (Fig. 5, Additional file 9: Figure S4, Additional file 10: Figure S5, and Additional file 11: Figure S6) suggest that the mean time of emergence of the ancestor for the major HEV genotypes infecting humans (Genotypes 1, 2, 3 and 4) ranged from 644 to 738 years ago. For genotypes 1and 2, their common ancestor emerged from 342 to 529 years ago; while the common ancestor of genotypes 3 and 4 emerged from 550 to 719 years ago. Separately, the respective common ancestors of genotypes 1, 3 and 4 emerged from 98 to 122 years ago, 248 to 265 years ago; and 145 to 161 years ago, respectively. In order to calculate the origin for the HEV genotypes 1, 2, 3 and 4, the strict clock model and the relaxed uncorrelated clock lognormal model were generated with an outgroup (rat HEV), while the relaxed uncorrelated clock exponential model was generated without the outgroup. The results revealed that in the three models, the root for HEV genotypes 1–4 falls between the strictly-human genotypes 1–2 and the zoonotic genotypes 3–4, reflecting, therefore, the transmission phenotypes of these four HEV genotypes.

In addition, the common ancestor for rat HEV genotypes and genotypes 1–4 was estimated to emerge from 6849 years ago (4112.00–9795.83) in strict clock model, and from 3911 years ago (2263.10–5817.47) in the uncorrelated clock model, with a divergence time between the two estimations of about 2937 years. These results also suggested that the HEV genotype 1 is the most recent compared to the zoonotic genotypes 3 and 4, with all genotype 1 lineages emerged in the early twentieth century (87–199 years ago).

The genotypes 5 and 6 shared the same common ancestor than genotype 4, that emerged in the early 1500s with a posterior probability PP = 1. This observation was noted in two of the models the strict and uncorrelated lognormal clock models, whereas the uncorrelated exponential clock model suggested that these three genotypes shared the same common ancestor but emerged later in the early 1800s. Concerning, the genotype 7 strain (isolated from camel), seems to share a common ancestor with genotype 3 strains. However, for genotypes 5, 6 and 7 only 1, 2 and 3 sequences respectively were included in this study, therefore the above observations should be taken with caution given the sampling bias.

## Discussion

In the present study, we analyzed the codon usage patterns and the evolutionary history of the three HEV coding sequences (ORF1, ORF2, and ORF3) to determine and shed some light on the factors governing their molecular evolution. To highlight genotype-specific patterns in the codon usage of HEV coding sequences, all the HEV sequences used by Smith et al. [9] for the most recent classification of HEV into 7 genotypes were analyzed in this study. Besides, three newly discovered HEV sequences in Bactrian camels (*Camelus bactrianus*), which have been proposed to form a new genotype (genotype 8) were also included [15].

The HEV genomes showed an A/C bias in the overall nucleotide composition with an overrepresentation of C. Such A/C bias was also reported in rubella virus, hepatitis C virus and GB-virus [16]. However, most of RNA positive- and negative-stranded viruses are biased towards a high prevalence of A rather than C [16]. The reason for these two opposite patterns in nucleotide bias is not clear, but as suggested previously, this bias could be the result of an adaptation of a common ancestor of modern HEV strains to the requirement, in terms of nucleotide composition, of the host during the evolutionary process [17]. For many organisms, including viruses, a GC- or AU-rich composition tends to correlate with their RSCU patterns. For instance, a GC- or AU-rich genome tends to contain codons preferentially ending with either G/C or A/U, respectively. Such trends, when present, support the influence of mutation pressure, as it is the case with ORF3 (Fig. 4). However, in ORF1 and ORF2, despite a higher percentage of GC versus AU, the RSCU analysis revealed a greater codon usage bias toward U terminated codons, suggesting thus other factors besides nucleotide composition that participate in shaping the synonymous codon usage in these two ORFs.

Next, we analyzed the ENC of the HEV ORFs to evaluate the extent of the codon usage bias in HEV genes. There was a significant difference in the overall bias between ORF1 and the other ORFs, with these latter being more biased. Such a difference in the degree of bias between individual genes in viruses that encode their proteins in separate open reading frames was previously reported [18]. The ORF1 high ENC value (52.8 ± 1.91) indicates that all possible codons are used with almost no preference, and similar values have been registered in other RNA viruses, like hepatitis C virus (ENC, 51.9) [19] and Zika virus (ENC, 53.93) [20]. Such low bias in the RNA viruses allows an efficient replication in the host cells by reducing the competition between the virus and the host for the synthesis machinery, especially for the genes needed in the early stages of infection (as it is the case with HEV ORF1). On the other hand, the codon usage bias was relatively higher in ORF2 (ENC, 48.62 ± 1.5) and ORF3 (ENC, 48.5 ± 3.6), but it remains lower than some RNA viruses such as the hepatitis A virus (ENC, 38.9) [19].

Mutation pressure rather than natural selection was identified as the most important determinant of codon bias in RNA viruses [18]. However, in some RNA viruses, it was demonstrated that natural selection exerted a predominant influence [20]. Concerning HEV, the results of ENC, PR2, and RSCU analyses pointed towards the influence of natural selection, which in accordance with recently published data [21], but in contradiction with an earlier study that concluded that the effects of mutation pressures were more important [17]. It is to note that in this latter study, besides the small number of the analyzed sequences, the parity rule 2 and neutrality plot analyses were not performed and the ENC-GC3s plots were constructed for the whole HEV genomes.

Further, we analyzed the codon adaptation of the HEV ORFs to different hosts independently of nucleotide content and amino acid composition using the normalized codon adaptation index [14]. The results showed that the adaptation of HEV strains to its host was ORF and genotype dependent. Interestingly, ORF1s and ORF2s were more adapted to *Macaca fascicularis* and humans more than any other host, indicating that the *Macaca fascicularis* could be a more suitable animal model for the study of HEV infection. Moreover, only the ORF1 of genotype 1 was significantly adapted to humans. The codon adaptation of virus is essential for fine-tuning the interaction with a given host [22], and the most affected genes by this mechanism are usually those highly needed in the early stages of the interaction [23]. Following this reasoning, the adaptive genetic changes observed in the NS1 gene of Zika virus were suggested as an explanation for the emergence of ZIKV in humans and the increase in viral fitness of the Asian lineages [24]. Similarly, therefore, the adaptation ORF1 of genotype 1 to humans could be considered as a strong indicator of an evolutionary adaptive change, which could be in turn associated with an improvement in translational efficiency and an increased the genotype 1 infectivity in humans. This may also explain the epidemic character of HEV genotype 1 infections. Moreover, the clustering of the HEV strains in the correspondence analysis and discriminant analyses (based on RSCU and N-CAI, respectively) was consistent with the genotypic classification based on HEV complete genomes proposed by Smith et al. [9]. Moreover, when the ORFs were analyzed separately, ORF1 and ORF3 were more efficient in separating the HEV strains into human and zoonotic clusters, indicating an evolutionary role of ORF1 and ORF3 as host species determinants [3, 17].

Further, we investigated the origin of HEV and the emergence of its genotypes using a Bayesian approach. Our estimation of the evolutionary rate based on the analysis of the 3′ end of HEV ORF1 (852 nt) ranged between 1 and 1.66 × 10–3 subs/site/year (strict and lognormal clock models). This speed of evolution was in the range reported in previous studies that analyzed the ORF2 genes used a similar Bayesian approach [25–27]. An earlier study, however, reported a slower rate (0.81–0.94) subs/site/year for the ORF1 RNA-dependent RNA polymerase coding segment), but they used a different approach based on linear regression analysis and maximum likelihood [28].

Herein, we dated the origin of the HEV genotypes 1, 2 3 and 4 the end of the thirteenth century, a date which falls within the previous estimates [27]. This date represents also the time where the common ancestor split into strictly-human and zoonotic genotypes. By introducing rat HEV sequences into the analysis, we traced back the common ancestor of all HEV sequences to ~ 6800 years ago, much greater than the estimates reported by Purdy et al. [27] but the range recently published by Forni and co-workers [29]. This period follows the time of appearance of human settlement and intensification of agricultural activities and domestication of pigs in East-Asia (China and neighboring regions) [30, 31]. In the Forni et al. study, the authors concluded that HEV originated from a human-infecting ancestor [29]. This raises a question why the all reported evolutionary models [27, 29] converged when estimating the recent apparition of human genotypes 1 and 2? If the host of the HEV ancestor was human and it spread later on to other species, then intuitively, it is more expected to find the human genotypes appearing earlier, and after adaptive changes, the other genotypes emerged. Besides in our analysis, not only the genotype 1 was the last to emerge but also a significant adaptive change to humans occurred in the ORF1 of only genotype 1. The domestication of animals occurs usually by separating them from the wild-life conditions which allow the occurrence of evolutionary adaptation to the new conditions that could in turn trigger changes in the related pathogens such as viruses. Therefore, it is safe to speculate that the domestication of pigs could have allowed the emergence of ancestor modern HEV from already existing strains in the wild animals.

In our analysis, the genotype 3 was the first to emerge followed by genotype 4, then genotype 2 and finally genotypes 1. Similar results were previously reported and discussed with minor divergence on the dates that can be explained by the number of sequences included, the fragment of the genome analyzed and the evolutionary models used [26, 27, 29, 32]. However, one divergence from other works, our findings indicated the genotype 7 (camel HEV) shared the same ancestor than genotype 3 while the genotypes 5 and 6 had a common ancestor with genotype 4, but given the sampling bias (only few sequences of genotypes 2, 5, 6 and 7 were included), the phylogeny estimates of these genotypes should be taken with great caution, and future isolation of strains belonging to these genotypes may yield different results.

## Conclusions

In conclusion, our results suggest that the common ancestor of the modern HEV strains emerged ~ 6800 years ago, in the period following the domestication of pigs and the intensification of agriculture. Under the domestication conditions, adaptive changes occurred in the common ancestor allowing the emergence of zoonotic and human HEV strains. Although mutation pressure effects cannot be excluded in the evolution process, as it has been documented in other RNA viruses, natural selection was identified as the major factor influencing the codon usage patterns in HEV ORFs, which may explain or could be explained by the wide range of HEV hosts. Interestingly, all these factors together permitted the occurrence of significant adaptation changes in the ORF1 of genotype 1 to humans, making the ORF1 an evolutionary indicator of HEV host speciation. However, further history or fossil record findings as well as the isolation of new HEV strains from more hosts are need for the determination of the accurate evolutionary history of HEV.

## Methods

### HEV sequences

The complete genomes of HEV strains used by Smith et al. [1] for the newly proposed classification of HEV isolates were retrieved from the National Center for Biotechnology Information (NCBI) database (http://www.ncbi.nlm.nih.gov). The sequences were aligned/edited/corrected using MAGA 7.0 and the alignments were further filtered to remove identical or highly similar sequences regardless of host or subtype. Only the open reading frames (ORFs) were considered for the analyses. The detailed information of the selected HEV complete genomes is listed in Additional file 1: Table S1.

### Compositional properties

Nucleotide compositional analysis of all HEV ORFs sequences was operated using CodonW program (http://codonw.sourceforge.net/). The overall frequency of occurrence of the nucleotides (A%, C%, T/U%, and G%), nucleotide occurrence at the 3rd codon position (U3%, G3%,C3% and A3%) and frequencies of occurrence of nucleotides G + C at the first (GC1S), second (GC2S), and third synonymous codon positions (GC3S) were calculated. Further, the mean frequency of G + C at GC1–2 positions and the total AU/GC contents were measured. The codons AUG and UGG are the only codons for Met and Trp, respectively, and the termination codons UAA, UAG, and UGA do not encode any amino acids. Therefore, these five codons are not expected to exhibit any usage bias and were therefore excluded from the analysis.

### Relative synonymous codon usage (RSCU) and correspondence analysis (COA)

The RSCU values for all of the coding sequences of HEV genomes were calculated to determine the characteristics of synonymous codon usage without the confounding influence of amino acid composition and coding sequence size of the different gene samples as was proposed by Sharp and Li in 1986 [33]. The RSCU index was calculated as follows:

$$ RSCU=\frac{Gij}{\sum_j^{ni} Gij} ni $$

Where *G*_*ij*_ represents the observed number of codons for the amino acid and *ni* represents the degenerate numbers of a specific synonymous codon that ranges from 1 to 61. The relative synonymous codon usage is the ratio of the observed frequency of a codon to the expected frequency of a codon if all the synonymous codons for a particular amino acid are used equally. Codons with RSCU values> 1.0 show positive codon usage bias and were defined as ‘abundant’ codons, whereas those with RSCU values < 1.0 show negative codon usage bias and were defined as ‘less-abundant’ codons. Codons with an RSCU value of *>* 1.6 were regarded as over-represented, while codons with an RSCU value of *<* 0.6 were regarded as under-represented. Codons used at an average level (no bias) have the RSCU values of 1 [34].

Correspondence analysis (COA) is a multivariate statistical method that is widely used to identify major sources of variation in synonymous codon usage among genes. In this study, the HEV individual coding region was represented as a 59-dimensional vector, and each dimension corresponds to the RSCU value of one sense codon (excluding Met, Trp, and the termination codons). Then, like principal components, the data were represented in a low-dimensional projection (2D display). Next, a Spearman’s rank correlation analysis was used to identify the relationship between nucleotide composition and the first two axes (Axis 1 and Axis 2) of the COA of HEV RSCU values. All statistical analyses were carried out using SPSS 17 (SPSS Inc., Chicago, IL, USA).

### Analysis effective number of codons (ENC)

The effective number of codons is a measure of bias from equal codon usage and its value is dependent upon the nucleotide composition of a gene. In this study, the ENC analysis was used to quantify the absolute codon usage bias by evaluating the degree of codon usage bias displayed by the HEV coding sequences, regardless of the gene lengths and the number of amino acids. The ENC can take values from 20, in the case of extreme bias where one codon is exclusively used for each amino acid, to 61 when the use of alternative synonymous codons is equally likely. The larger the extent of codon preference in a gene, the smaller the ENC value. It is also generally believed that genes have a significant codon bias when the ENC value is less than or equal to 35 [34]. The ENC was calculated according to the following formula:

$$ ENC=2+\frac{9}{F2}+\frac{1}{F3}+\frac{5}{F4}+\frac{3}{F6} $$

Where F (*i* = 2, 3, 4, 6) represents the mean values of F*i* with *i*-fold degenerate amino acids. The F*i* was calculated according to the equation below:

$$ Fi=\frac{n{\sum}_{j=1}^i{\left(\frac{nj}{n}\right)}^2-1}{n-1} $$

To statistically analyze the ENC values of the HEV ORFs among the different genotypes, a one-way ANOVA was used to compare the groups’ means. Besides, given that the homogeneity of variances was violated, the Welsh and Brown-Forsythe tests were run to compare the means. Then, Games-Howell post hoc test was adopted for multiple-comparison of the ENC values between the different genotypes. The genotypes 2 and 5, were excluded from the analysis because only one sequence for each of these genotypes was included in the study; genotypes 6, 7 and 8 were included in testing the inequality of the means but only genotypes 1, 3 and 4 were included in the multiple-comparison test.

### Analysis of the codon adaptation index (CAI)

The CAI is a universal measure of codon bias of genes in different organisms. It measures the deviation of a given protein-coding gene sequence with respect to a reference set of genes [35]. The CAI values range from 0 to 1where the most frequent codons have the highest relative adaptiveness values, and sequences with higher CAIs are preferred over those with lower CAIs. Comparative analysis of the codon usage was implemented between HEV genes and viral hosts: humans (*Homo sapiens*), Cynomolgus monkey (*Macaca fascicularis*), rhesus monkey (*Macaca mulatta*), wild-boar (*Sus scrofa*), pig (*Sus scrofa* domestica), rabbit (*Oryctolagus cuniculus*) and camel (*Camelus dromedarius* and *Camelus bactrianus*). Codon usage tables for host genes were downloaded from the codon usage Kazusa database (http://www.kazusa.or.jp/codon/). Moreover, in order to provide statistical support to CAI analyses. The expected CAI value (E-CAI) for the three HEV ORFs coding sequences was calculated using E-CAL server [14]. A Kolmogorov–Smirnov test was used to calculate E-CAI values at 95% confidential interval. Then, the normalized CAI value (N-CAI), which is defined as the quotient between the CAI for each gene and its expected value, was also calculated. An N-CAI value greater than one indicates that the observed CAI is bigger than its expected value, which could be interpreted as the result of a statistically significant adaptation process in the codon usage [14]. Nonsynonymous codons and termination codons were excluded from the calculation.

### Similarity index analysis

In order to calculate the influence of complete codon usage of the main hosts on HEV ORFs codon usage, the similarity index analysis was carried out. The calculation was performed as previously described [20] according to the following equations:

$$ R\left(A,B\right)=\frac{\sum_{i=1}^{59}{a}_i\times {b}_i}{\sqrt{\sum_{i=1}^{59}{a_i}^2\times {\sum}_{i=1}^{59}{b_i}^2}} $$

$$ D\left(A,B\right)=\frac{1-R\left(A,B\right)}{2} $$

As R(A, B) determined the value of cosine that has an angle between A and B spatial vectors, shows similarity level among HEV ORFs and hosts general codon usage pattern. The (a_i_) represents RSCU value for a specific codon among all the synonymous codons, whereas (b_i_) is the RSCU value for the same codon of the host. D(A, B) shows the possible effect of the host overall codon usage on HEV ORFs with a value ranging from 0 to 1.0. Codon usage tables of the hosts were retrieved from the Codon Usage Database (http://www.kazusa.or.jp/codon/).

### Effect of mutation pressure and translational selection on the codon usage pattern of HEV ORFs

To determine the influence of nucleotide compositional constraint on structuring synonymous codon usage bias, the observed and expected ENC values were compared in an ENC-GC3s plot. In such a plot, if the observed data points are on or nearby the null expected ENC curve, then compositional constraint plays the major role in structuring codon usage patterns. If the observed points are fallen below the null curve, then the role of translational (natural) selection emerges. For revealing the relationship between GC3s and ENC values, the expected ENC values for different GC3s were calculated as follows:

$$ ENCexpected=2+s+\left(\frac{29}{s^2+\left(1-{s}^2\right)}\right) $$

where (s) represents the given GC3s content [35].

Further, analysis of the correlation between the GC contents at the first and second codon positions (GC12) and that at the third codon position (GC3) is useful to investigate the varying roles of mutational pressure and natural selection in shaping the codon usage bias of HEV genes. Therefore, the GC3 was plotted against the GC12 in a neutrality plot [36] and analyzed as previously described [37]: if the correlation between GC12 and GC3 is significant, the mutation pressure is regarded as the main force forming the codon usage bias.

Next, we used the Parity rule 2 (PR2) plot to assess the influence of mutation pressure and translational selection on the codon usage of the HEV genes [38]. The GC-bias [G3/(G3 + C3)] at the third codon position of the four-codon amino acids (alanine, arginine, glycine, leucine, proline, serine, threonine and valine) of the entire genes was plotted against the AU-bias [A3/(A3 + U3)] at the same codon position of the same amino acids. The center of the plot, where both coordinates are 0.5, is the place where A = U and G = C (PR2), with no biases between the influence of mutation and selection rates (substitution rates) [38].

### Bayesian coalescent Markov chain Monte Carlo (MCMC) analysis

A total of 183 HEV sequences with known collection dates (ranging from 1982 to 2017), host and region (samples from 25 different countries) were obtained from GenBank (Additional file 1: Table S1), including 28 genotype 1 sequences, 2 genotype 2 sequences, 81 genotype 3 sequences, 55 genotype 4 sequences and 17 sequences with unassigned genotype. Next, the ORF1 sequences were aligned using MEGAX software [39], manually controlled and adjusted by visual inspection, and then cropped to the same length (852 nt corresponding to nt 4270–5121 in the genotype 4 reference strain AB197673) using Bioedit software (freely available at http://www.mbio.ncsu.edu/bioedit/bioedit.html). The sequence alignments are available from the authors upon request.

After alignment, the Bayesian phylogenetics analysis was performed. The age of the most recent common ancestor (tMRCA) was estimated using the Bayesian Markov Chain Monte Carlo (MCMC) statistical framework implemented in BEAST v1.10.4 package [13, 40, 41]. All the calculation were carried out under the GTR + I + Γ4 nucleotide substitution model, with the rate of each substitution type under the general reversible model (GTR), the proportion of invariant sites (I), and shape parameter of a gamma distribution with four rate categories (Γ4). Using a constant size coalescent prior as tree prior, strict molecular clock model, assuming a single evolutionary rate for each branch of the tree, versus relaxed uncorrelated models (lognormal and exponential), in which the rate of evolution is allowed to vary among branches, were compared. A uniform prior was applied on the clock rate (0.8–1.8 × 10–3, initial value: 1.35 × 10–3, subs./site/year) on the basis of estimations reported from previous studies [25, 27, 42].

The MCMC analysis was run until convergence that was assessed by estimating the effective sampling size (ESS > 200) after a 10% burn-in, using Tracer software v1.7.1 (included in the BEAST package), and the uncertainty of the estimates was indicated by 95% highest posterior density (95% HPD) intervals. The best-fitting models were selected using a Bayes factor based on marginal likelihoods implemented in BEAST [43, 44]. The phylogenetic trees were summarized using TreeAnnotator v1.10.4 and visualized with FigTree v1.4.4, both included in the BEAST package.

## Supplementary information

**Additional file 1: Table S1.** Information of the selected HEV sequences included in the study.

**Additional file 2: Table S2.** Nucleotide composition of the HEV coding sequences.

**Additional file 3: Tables S3** and **S4.** RSCU patterns of the HEV coding sequences, sorted by ORF and by genotype. Detailed information on common vs uncommon preferred codons, ratio of common preferred codons and over- vs under-represented preferred codons in the HEV coding sequences.

**Additional file 4: Table S5.** Normalized codon adaptation index (N-CAI) of HEV ORFs.

**Additional file 5: Table S6.** Group membership prediction according to the discriminant function obtained in discriminant analysis based on the normalized codon adaptation index (N-CAI) of the HEV ORFs in relation to all the hosts.

**Additional file 6: Figure S1.** Analysis of the similarity index of the codon usage between HEV strains and its main hosts. All three HEV ORFs were analyzed together regardless of the genotype and the data were colored according to the ORF (A). Then, the ORF1s, 2 s and 3 s were analyzed separately and the data were colored according to the different genotypes (B, C and D, respectively). A series of two-way ANOVA was performed using the Host as the first independent nominal variable and ORFs regardless of the genotype (A), ORF1 (B), ORF2 (C) or ORF3 (D) as the second independent nominal variables, followed by Bonferroni’ post hoc test. For the ORFs variable all the combination in multiple comparison test were significant (*p* < 0.001), while for the Host variable the results are shown in the Figure (A). In (B), (C) and (D), the difference between the different hosts was statistically significant (not shown), while the results for the ORF1, ORF2 and ORF3 variables are presented in the figure. Given the few sequences of genotypes 2, 5, 6, 7 and 8, the statistical analysis was performed only on the sequences of genotypes 1, 3 and 4. The data are presented as mean ± standard error; *p < 0.05, **p < 0.01, ***p < 0.001; ns: non-significant *p* > 0.05.

**Additional file 7: Figure S2.** Parity rule 2 (PR2) bias plot [A3/(A3 + U3) against G3/(G3+ C3)]. PR2 plots were constructed for all three HEV ORFs together (A), and for the ORF1s, 2 s and 3 s separately (B, C and D, respectively).

**Additional file 8: Figure S3.** *P*-value plot against P3. G + C contents of the first codon position P1, G + C contents of the second codon position P2*,* neutrality plots (GC_1,2S_ (P1,2) and that of the third codon position (GC_3S,_ P3) were constructed for all three HEV ORFs and individual HEV ORFs.

**Additional file 9: Figure S4.** Detailed Bayesian phylogenetic maximum clade credibility (MCC) tree for 183 sequences of HEV ORF1 (852 nt of the 3′ end). This tree was constructed using a strict clock model with a constant growth prior. The numbers at each tree represent the mean values for age of the most recent common ancestor (MRCA) at that node. Each sequence is labeled with its year of collection followed by GenBank accession number, genotype, region of isolation and the host.

**Additional file 10: Figure S5.** Detailed Bayesian phylogenetic maximum clade credibility (MCC) tree for 183 sequences of HEV ORF1 (852 nt of the 3′ end). This tree was constructed using an uncorrelated relaxed clock model with a lognormal growth prior. The numbers at each tree represent the mean values for age of the most recent common ancestor (MRCA) at that node. Each sequence is labeled with its year of collection followed by GenBank accession number, genotype, region of isolation and the host.

**Additional file 11: Figure S6.** Detailed Bayesian phylogenetic maximum clade credibility (MCC) tree for 183 sequences of HEV ORF1 (852 nt of the 3′ end). This tree was constructed using an uncorrelated relaxed clock model with an exponential growth prior. The numbers at each tree represent the mean values for age of the most recent common ancestor (MRCA) at that node. Each sequence is labeled with its year of collection followed by GenBank accession number, genotype, region of isolation and the host.

## Data Availability

The datasets used and analyzed in the current study are available from the corresponding author on reasonable request.

## Abbreviations

A, C, G, T and U: Adenine, cytosine, guanine, thymine and uracil
aa: Amino acid
CAI: Codon adaptation index
COA: Correspondence analysis
E-CAI: Expected codon adaptation index
ENC: Effective number of codons
HEV: Hepatitis E virus
MCMC: Coalescent Markov Chain Monte Carlo
N-CAI: Normalized codon adaptation index
nt: Nucleotide
ORF(s): Open reading frame(s)
PP: Posterior probability
PR2 plot: Parity rule 2 plot
RSCU: Relative synonymous codon usage
tMRCA: Time to the most recent common ancestor
