# Supplementary material for: Comprehensive analysis of genetic and evolutionary features of the hepatitis E virus
Source: BMC Genomics. 2019 Oct 29;20:790. doi: 10.1186/s12864-019-6100-8 (PMC6820953; doi:10.1186/s12864-019-6100-8)
Supplement: Supplementary file 4 — Additional file 4: Table S5. Normalized codon adaptation index (N-CAI) of HEV ORFs. [file 12864_2019_6100_MOESM4_ESM.docx]

**Table S5. Normalized codon adaptation index (N-CAI) of HEV ORFs**

|  | **ORF1** |  |  |  |  |  |  |  |
| --- | --- | --- | --- | --- | --- | --- | --- | --- |
|  | **G1** | **G2** | **G3** | **G4** | **G5** | **G6** | **G7** | **G8** |
| ***Homo sapiens*** | **1.017** | 0.995 | 0.997 | 0.993 | 0.996 | 0.990 | 0.986 | 0.995 |
| ***Oryctolagus cuniculus*** | 0.996 | 0.973 | 0.976 | 0.969 | 0.976 | 0.977 | 0.970 | 0.969 |
| ***Macaca fascicularis*** | **1.025** | **1.012** | **1.004** | **1.002** | 0.999 | 0.998 | 0.996 | 0.999 |
| ***Macaca mulatta*** | **1.015** | 0.988 | 0.994 | 0.990 | 0.994 | 0.994 | 0.988 | 0.986 |
| ***Sus scrofa*** | **1.007** | 0.981 | 0.988 | 0.981 | 0.980 | 0.983 | 0.979 | 0.981 |
| ***Sus scrofa domestica*** | 0.949 | 0.925 | 0.940 | 0.933 | 0.944 | 0.938 | 0.928 | 0.922 |
| ***Camelus dromedarius*** | **1.005** | 0.972 | 0.983 | 0.974 | 0.975 | 0.976 | 0.974 | 0.978 |
| ***Camelus bactrianus*** | 0.999 | 0.967 | 0.979 | 0.966 | 0.972 | 0.971 | 0.968 | 0.969 |
|  | **ORF2** |  |  |  |  |  |  |  |
|  | **G1** | **G2** | **G3** | **G4** | **G5** | **G6** | **G7** | **G8** |
| ***Homo sapiens*** | **1.002** | **1.007** | **1.008** | **1.003** | **1.024** | 0.998 | 0.980 | 0.993 |
| ***Oryctolagus cuniculus*** | 0.977 | 0.989 | 0.984 | 0.978 | 0.995 | 0.974 | 0.956 | 0.971 |
| ***Macaca fascicularis*** | **1.008** | **1.023** | **1.015** | **1.012** | **1.038** | **1.001** | 0.984 | **1.003** |
| ***Macaca mulatta*** | **1.001** | **1.007** | **1.006** | **1.004** | **1.030** | 0.999 | 0.974 | 0.994 |
| ***Sus scrofa*** | 0.995 | 0.997 | 0.998 | 0.992 | **1.003** | 0.990 | 0.965 | 0.991 |
| ***Sus scrofa domestica*** | 0.935 | 0.956 | 0.937 | 0.929 | 0.982 | 0.947 | 0.892 | 0.906 |
| ***Camelus dromedarius*** | 0.996 | **1.013** | 0.996 | 0.989 | **1.015** | 0.989 | 0.970 | 0.987 |
| ***Camelus bactrianus*** | 0.979 | 0.977 | 0.978 | 0.971 | 0.994 | 0.954 | 0.947 | 0.969 |
|  | **ORF3** |  |  |  |  |  |  |  |
|  | **G1** | **G2** | **G3** | **G4** | **G5** | **G6** | **G7** | **G8** |
| ***Homo sapiens*** | 0.906 | 0.974 | 0.969 | 0.926 | 0.905 | 0.930 | 0.952 | 0.935 |
| ***Oryctolagus cuniculus*** | 0.901 | 0.967 | 0.958 | 0.921 | 0.890 | 0.913 | 0.935 | 0.926 |
| ***Macaca fascicularis*** | 0.907 | 0.978 | 0.971 | 0.934 | 0.904 | 0.937 | 0.953 | 0.939 |
| ***Macaca mulatta*** | 0.896 | 0.976 | 0.962 | 0.929 | 0.898 | 0.922 | 0.942 | 0.932 |
| ***Sus scrofa*** | 0.903 | 0.977 | 0.963 | 0.926 | 0.894 | 0.923 | 0.943 | 0.933 |
| ***Sus scrofa domestica*** | 0.884 | 0.913 | 0.983 | 0.924 | 0.863 | 0.896 | 0.922 | 0.947 |
| ***Camelus dromedarius*** | 0.907 | 0.985 | 0.954 | 0.923 | 0.907 | 0.928 | 0.937 | 0.927 |
| ***Camelus bactrianus*** | 0.916 | 0.996 | 0.957 | 0.922 | 0.906 | 0.922 | 0.936 | 0.931 |

^The values written in bold indicate statistically significant adaptation in the codon usage^
