# Supplementary material for: Comprehensive analysis of genetic and evolutionary features of the hepatitis E virus
Source: BMC Genomics. 2019 Oct 29;20:790. doi: 10.1186/s12864-019-6100-8 (PMC6820953; doi:10.1186/s12864-019-6100-8)
Supplement: Supplementary file 5 — Additional file 5: Table S6. Group membership prediction according to the discriminant function obtained in discriminant analysis based on the normalized codon adaptation index (N-CAI) of the HEV ORFs in relation to all the hosts. [file 12864_2019_6100_MOESM5_ESM.docx]

**Table S. Group membership prediction according to the discriminant function obtained in discriminant analysis based on the normalized codon adaptation index (N-CAI) of the HEV ORFs in relation to all the hosts.**

| **1. All ORFs** | | | |  |  |  |  |  |  |
| --- | --- | --- | --- | --- | --- | --- | --- | --- | --- |
|  | **Predicted Group Membership** | | | **Total** |  |  |  |  |  |
| **ORFs** | **ORF1s** | **ORF2s** | **ORF3s** |  |  |  |  |  |  |
| **ORF1s** | 83.3 | 16.7 | 0 | 100 |  |  |  |  |  |
| **ORF2s** | 16.9 | 83.1 | 0 | 100 |  |  |  |  |  |
| **ORF3s** | 0.6 | 0 | 99.4 | 100 |  |  |  |  |  |
| **2. ORF1s** |  |  |  |  |  |  |  |  |  |
|  | **Predicted Group Membership** | | | | | | | | **Total** |
| **Genotype** | **G1** | **G2** | **G3** | **G4** | **G5** | **G6** | **G7** | **G8** |  |
| **G1** | 100 | 0 | 0 | 0 | 0 | 0 | 0 | 0 | 100 |
| **G2** | 0 | 0 | 0 | 100 | 0 | 0 | 0 | 0 | 100 |
| **G3** | 1.3 | 0 | 84.8 | 11.4 | 0 | 1.3 | 0 | 1.3 | 100 |
| **G4** | 0 | 0 | 20.4 | 79.6 | 0 | 0 | 0 | 0 | 100 |
| **G5** | 0 | 0 | 100 | 0 | 0 | 0 | 0 | 0 | 100 |
| **G6** | 0 | 0 | 100 | 0 | 0 | 0 | 0 | 0 | 100 |
| **G7** | 0 | 0 | 0 | 66.7 | 0 | 0 | 33.3 | 0 | 100 |
| **G8** | 0 | 0 | 33.3 | 66.7 | 0 | 0 | 0 | 0 | 100 |
| **3. ORF2s** |  |  |  |  |  |  |  |  |  |
|  | **Predicted Group Membership** | | | | | | | | **Total** |
| **Genotype** | **G1** | **G2** | **G3** | **G4** | **G5** | **G6** | **G7** | **G8** |  |
| **G1** | 45.8 | 0 | 50 | 4.2 | 0 | 0 | 0 | 0 | 100 |
| **G2** | 0 | 0 | 100 | 0 | 0 | 0 | 0 | 0 | 100 |
| **G3** | 14.1 | 0 | 67.9 | 15.4 | 0 | 1.3 | 0 | 1.3 | 100 |
| **G4** | 0 | 0 | 41.7 | 58.3 | 0 | 0 | 0 | 0 | 100 |
| **G5** | 0 | 0 | 100 | 0 | 0 | 0 | 0 | 0 | 100 |
| **G6** | 0 | 0 | 50 | 50 | 0 | 0 | 0 | 0 | 100 |
| **G7** | 0 | 0 | 0 | 33.3 | 0 | 0 | 66.7 | 0 | 100 |
| **G8** | 33.3 | 0 | 33.3 | 33.3 | 0 | 0 | 0 | 0 | 100 |
| **4. ORF3s** |  |  |  |  |  |  |  |  |  |
|  | **Predicted Group Membership** | | | | | | | | **Total** |
| **Genotype** | **G1** | **G2** | **G3** | **G4** | **G5** | **G6** | **G7** | **G8** |  |
| **G1** | 95.8 | 0 | 0 | 4.2 | 0 | 0 | 0 | 0 | 100 |
| **G2** | 0 | 100 | 0 | 0 | 0 | 0 | 0 | 0 | 100 |
| **G3** | 0 | 0 | 92.3 | 6.4 | 0 | 0 | 1.3 | 0 | 100 |
| **G4** | 2 | 0 | 6.1 | 89.8 | 0 | 0 | 2 | 0 | 100 |
| **G5** | 0 | 0 | 0 | 0 | 100 | 0 | 0 | 0 | 100 |
| **G6** | 50 | 0 | 0 | 0 | 0 | 50 | 0 | 0 | 100 |
| **G7** | 0 | 0 | 0 | 50 | 0 | 0 | 50 | 0 | 100 |
| **G8** | 0 | 0 | 66.7 | 33.3 | 0 | 0 | 0 | 0 | 100 |

^All values are expressed as percentages^
