# Supplementary figures and images for: Comprehensive analysis of genetic and evolutionary features of the hepatitis E virus
Source: BMC Genomics. 2019 Oct 29;20:790. doi: 10.1186/s12864-019-6100-8 (PMC6820953; doi:10.1186/s12864-019-6100-8)

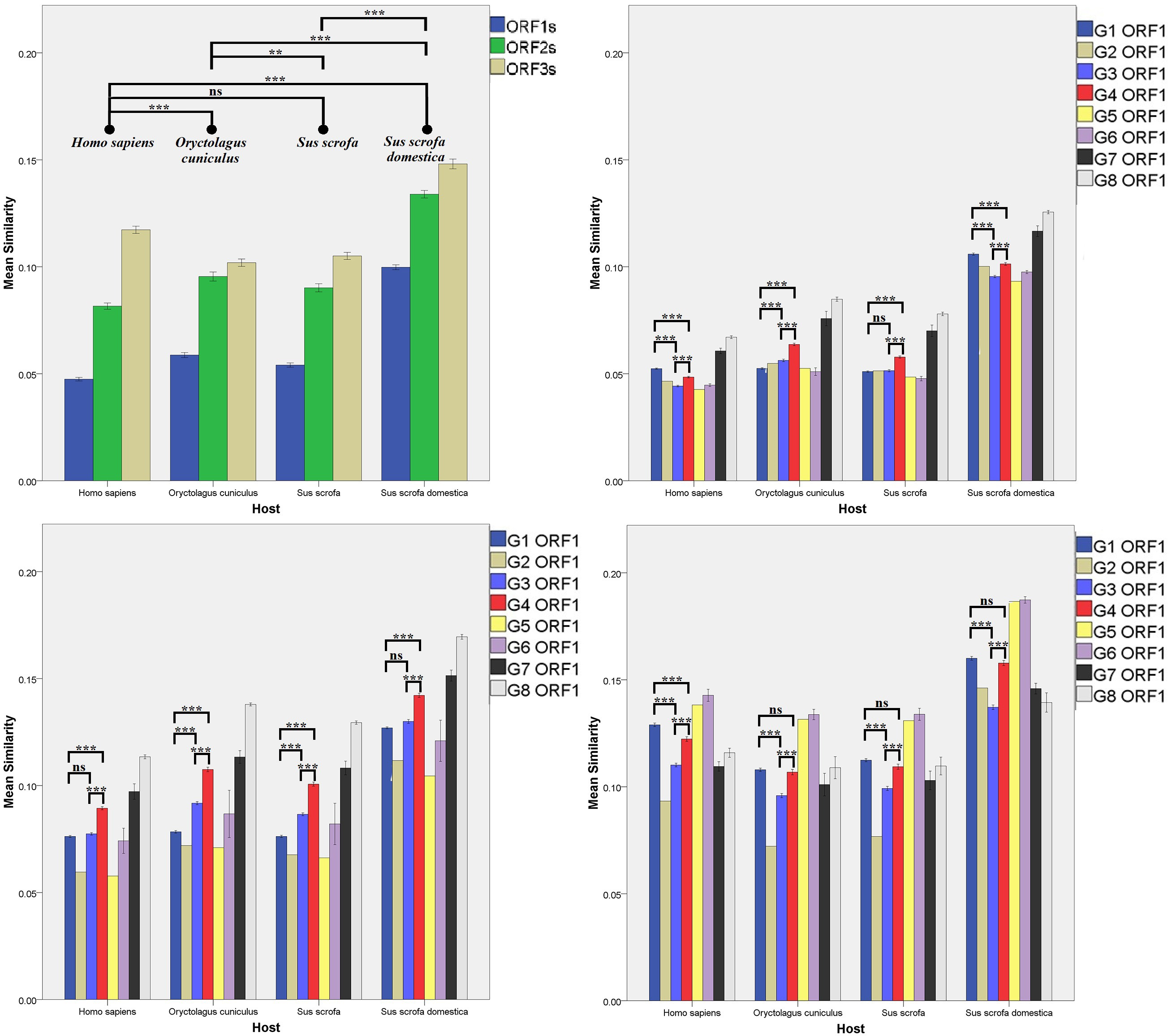

Supplement: Supplementary file 6 — Additional file 6: Figure S1. Analysis of the similarity index of the codon usage between HEV strains and its main hosts. All three HEV ORFs were analyzed together regardless of the genotype and the data were colored according to the ORF (A). Then, the ORF1s, 2 s and 3 s were analyzed separately and the data were colored according to the different genotypes (B, C and D, respectively). A series of two-way ANOVA was performed using the Host as the first independent nominal variable and ORFs regardless of the genotype (A), ORF1 (B), ORF2 (C) or ORF3 (D) as the second independent nominal variables, followed by Bonferroni’ post hoc test. For the ORFs variable all the combination in multiple comparison test were significant (p < 0.001), while for the Host variable the results are shown in the Figure (A). In (B), (C) and (D), the difference between the different hosts was statistically significant (not shown), while the results for the ORF1, ORF2 and ORF3 variables are presented in the figure. Given the few sequences of genotypes 2, 5, 6, 7 and 8, the statistical analysis was performed only on the sequences of genotypes 1, 3 and 4. The data are presented as mean ± standard error; *p < 0.05, **p < 0.01, ***p < 0.001; ns: non-significant p > 0.05. [file 12864_2019_6100_MOESM6_ESM.tif]

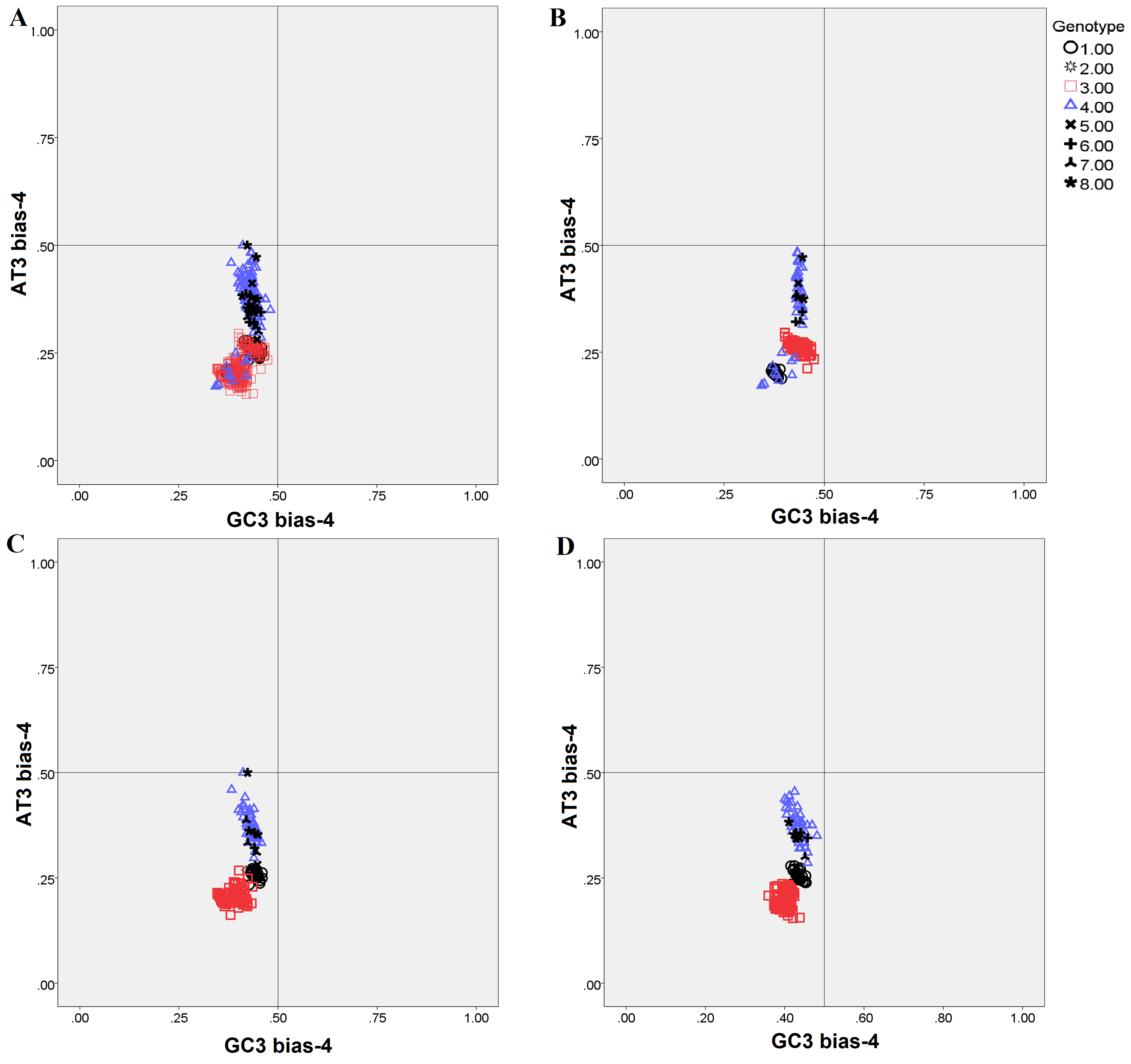

Supplement: Supplementary file 7 — Additional file 7: Figure S2. Parity rule 2 (PR2) bias plot [A3/(A3 + U3) against G3/(G3+ C3)]. PR2 plots were constructed for all three HEV ORFs together (A), and for the ORF1s, 2 s and 3 s separately (B, C and D, respectively). [file 12864_2019_6100_MOESM7_ESM.tif]

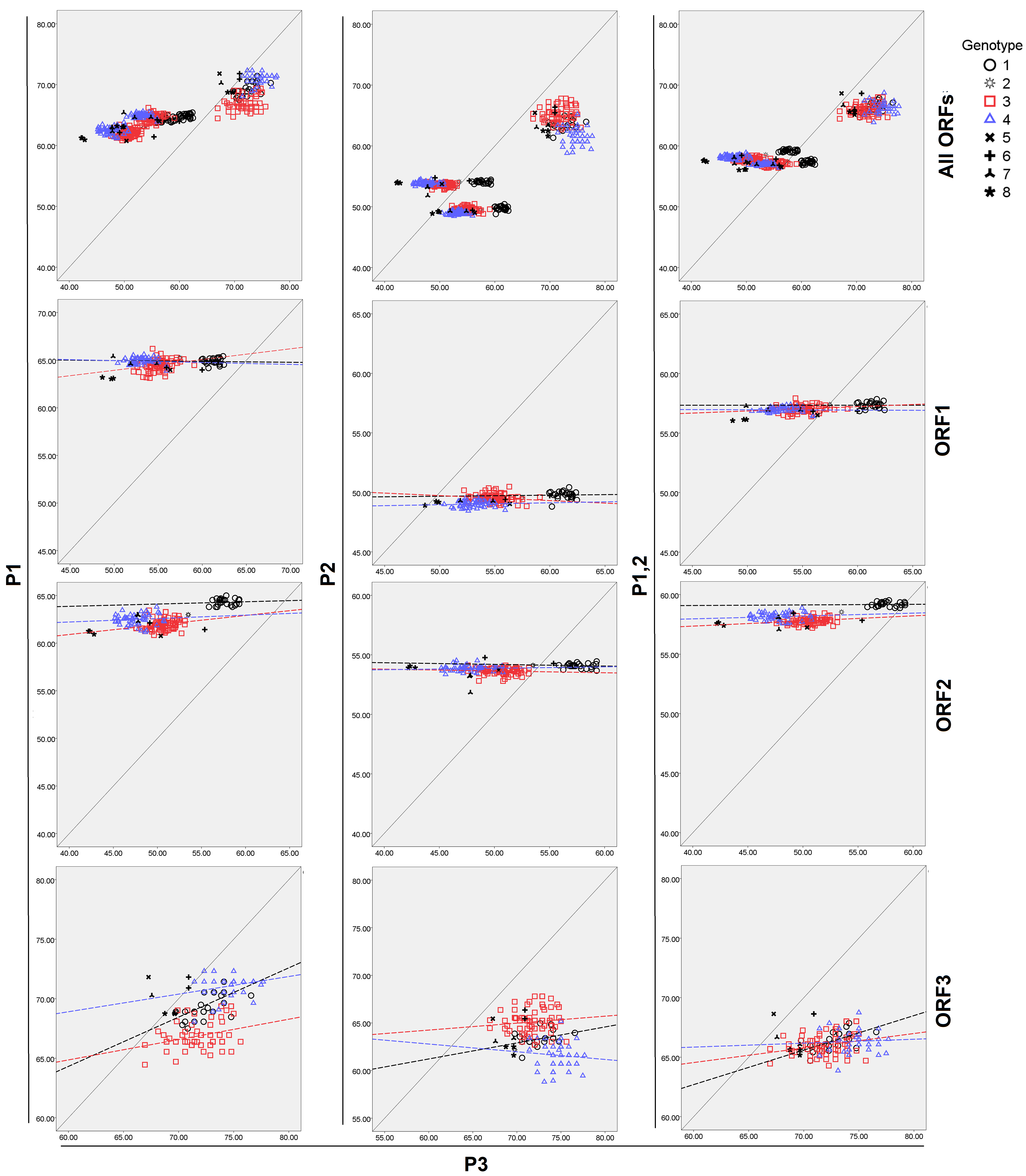

Supplement: Supplementary file 8 — Additional file 8: Figure S3. P-value plot against P3. G + C contents of the first codon position P1, G + C contents of the second codon position P2, neutrality plots (GC1,2S (P1,2) and that of the third codon position (GC3S, P3) were constructed for all three HEV ORFs and individual HEV ORFs. [file 12864_2019_6100_MOESM8_ESM.tif]

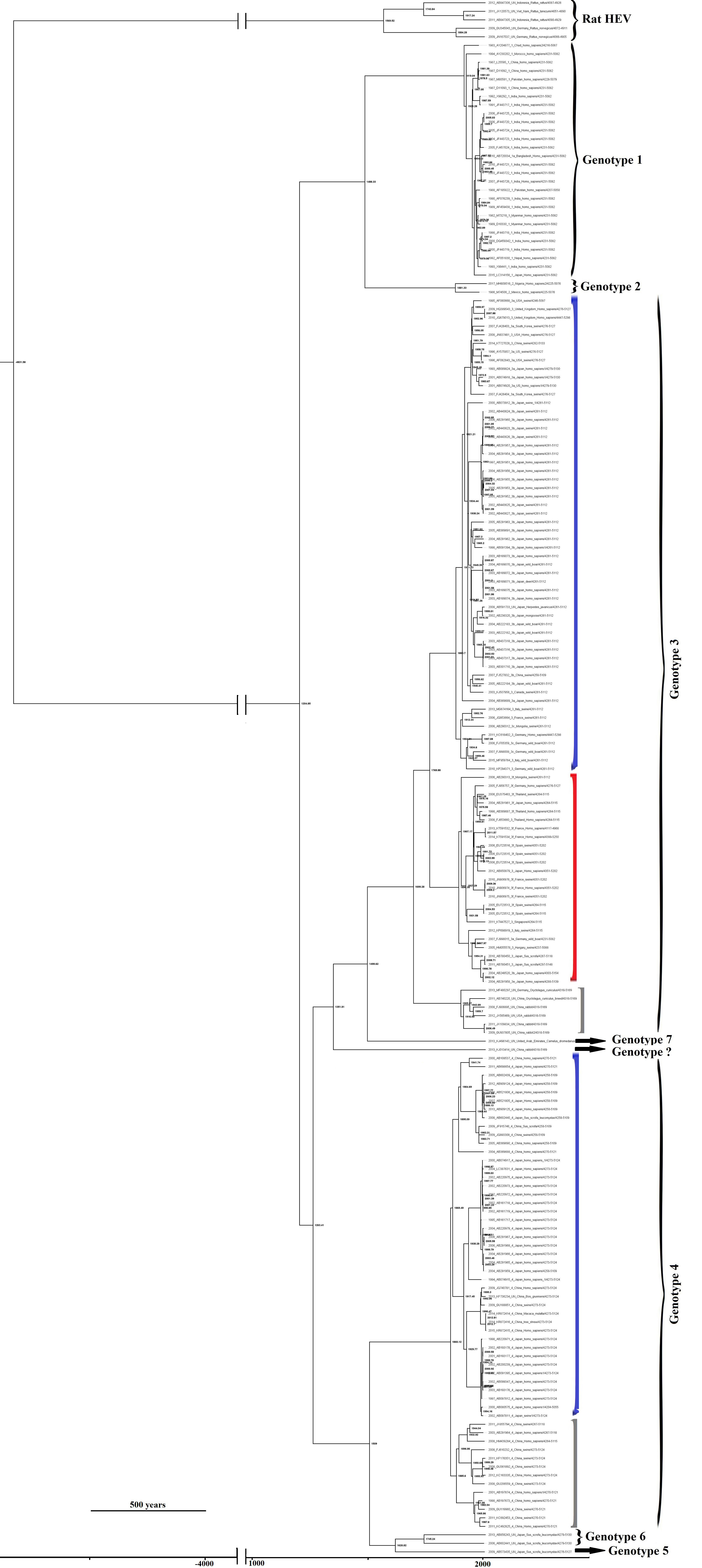

Supplement: Supplementary file 9 — Additional file 9: Figure S4. Detailed Bayesian phylogenetic maximum clade credibility (MCC) tree for 183 sequences of HEV ORF1 (852 nt of the 3′ end). This tree was constructed using a strict clock model with a constant growth prior. The numbers at each tree represent the mean values for age of the most recent common ancestor (MRCA) at that node. Each sequence is labeled with its year of collection followed by GenBank accession number, genotype, region of isolation and the host. [file 12864_2019_6100_MOESM9_ESM.tif]

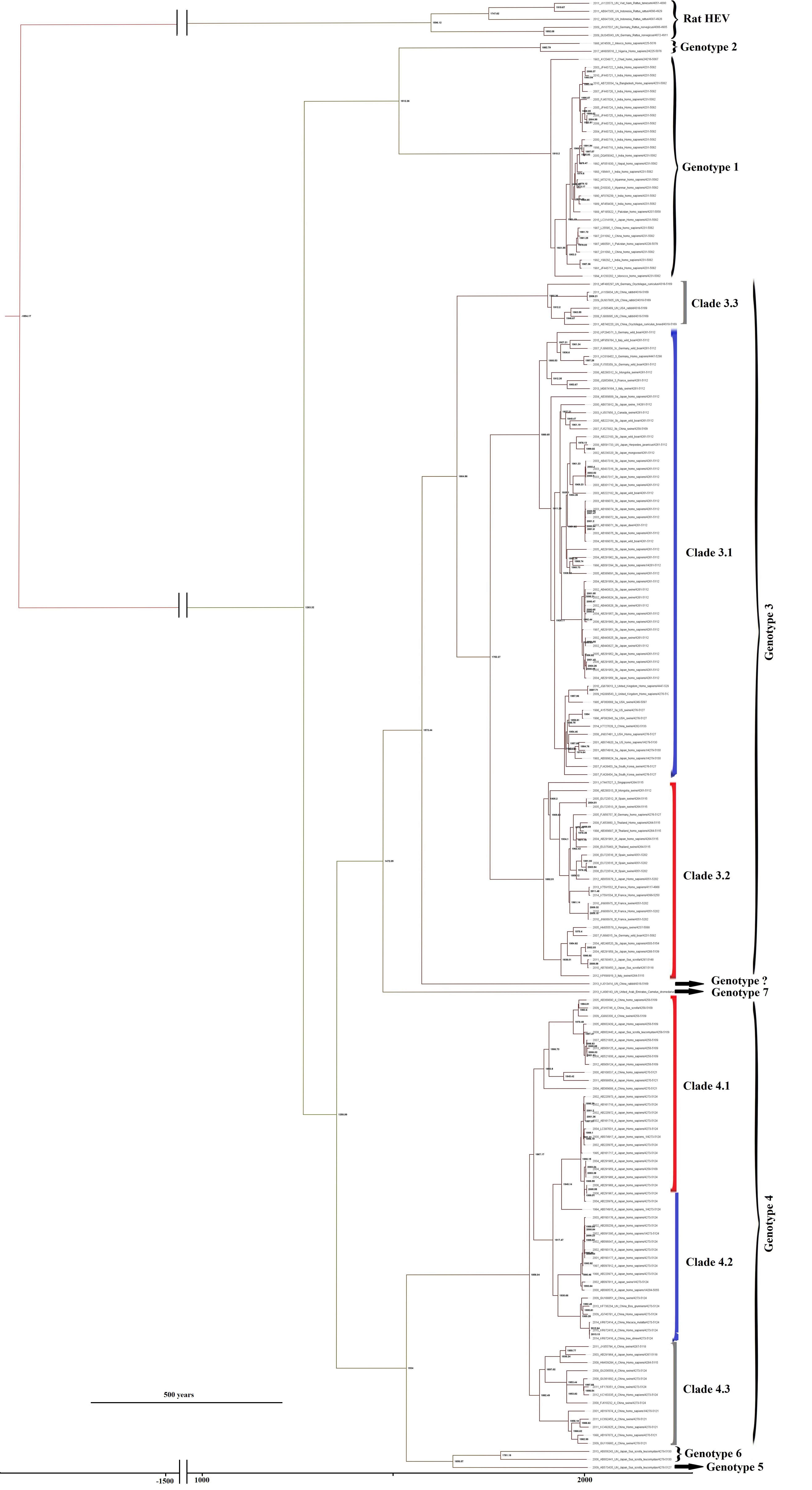

Supplement: Supplementary file 10 — Additional file 10: Figure S5. Detailed Bayesian phylogenetic maximum clade credibility (MCC) tree for 183 sequences of HEV ORF1 (852 nt of the 3′ end). This tree was constructed using an uncorrelated relaxed clock model with a lognormal growth prior. The numbers at each tree represent the mean values for age of the most recent common ancestor (MRCA) at that node. Each sequence is labeled with its year of collection followed by GenBank accession number, genotype, region of isolation and the host. [file 12864_2019_6100_MOESM10_ESM.tif]

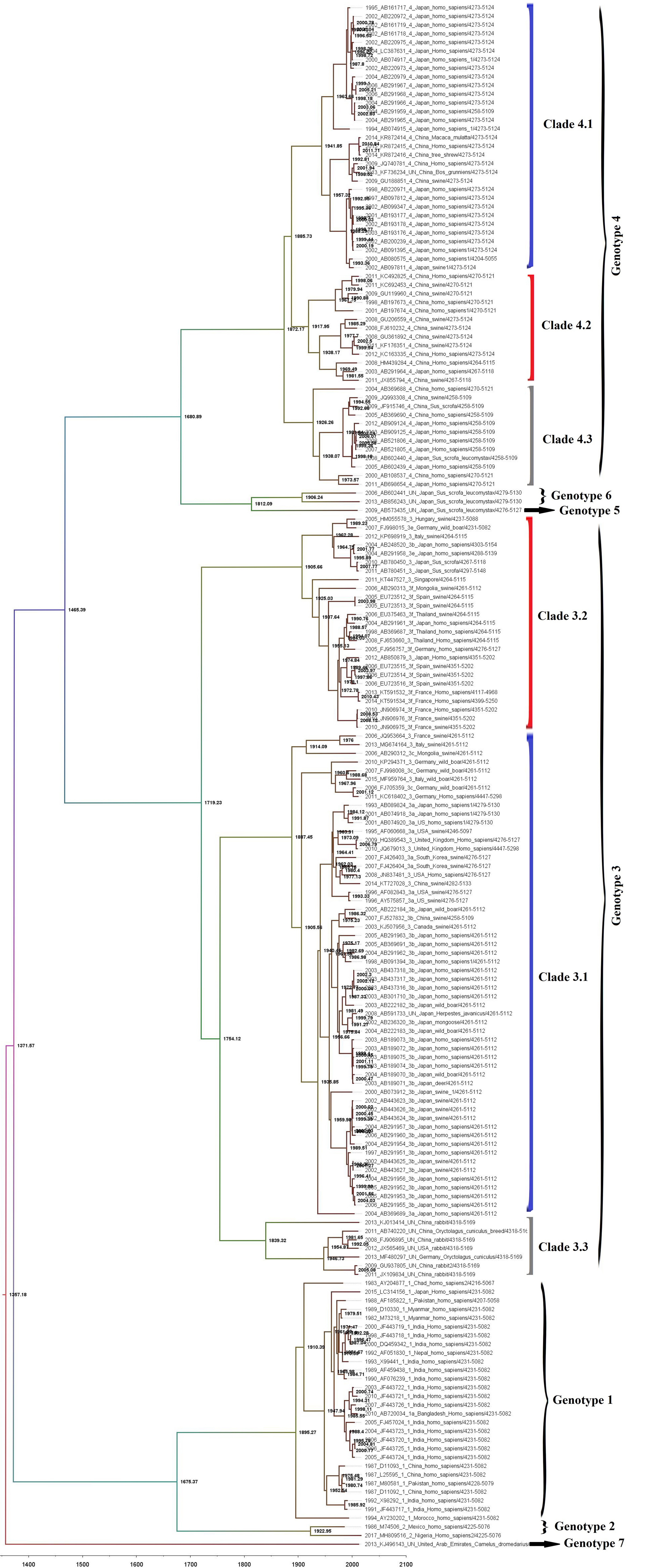

Supplement: Supplementary file 11 — Additional file 11: Figure S6. Detailed Bayesian phylogenetic maximum clade credibility (MCC) tree for 183 sequences of HEV ORF1 (852 nt of the 3′ end). This tree was constructed using an uncorrelated relaxed clock model with an exponential growth prior. The numbers at each tree represent the mean values for age of the most recent common ancestor (MRCA) at that node. Each sequence is labeled with its year of collection followed by GenBank accession number, genotype, region of isolation and the host. [file 12864_2019_6100_MOESM11_ESM.tif]
